# Novel insights into SLC25A46-related pathologies in a genetic mouse model

**DOI:** 10.1371/journal.pgen.1006656

**Published:** 2017-04-04

**Authors:** Maria Eirini Terzenidou, Aikaterini Segklia, Toshimi Kano, Florentia Papastefanaki, Alexandros Karakostas, Maria Charalambous, Fotis Ioakeimidis, Maria Papadaki, Ismini Kloukina, Margarita Chrysanthou-Piterou, Martina Samiotaki, George Panayotou, Rebecca Matsas, Eleni Douni

**Affiliations:** 1 Laboratory of Genetics, Department of Biotechnology, Agricultural University of Athens, Athens, Greece; 2 Biomedical Sciences Research Center "Alexander Fleming", Vari, Greece; 3 Laboratory of Cellular and Molecular Neurobiology, Hellenic Pasteur Institute, Athens, Greece; 4 Center of Basic Research, Biomedical Research Foundation of the Academy of Athens, Athens, Greece; 5 Laboratory of Neurobiology, Histochemistry and Electron Microscopy, 1st Department of Psychiatry, University of Athens Medical School, Eginition Hospital, Athens, Greece; Dunn Human Nutrition Unit, UNITED STATES

## Abstract

The mitochondrial protein SLC25A46 has been recently identified as a novel pathogenic cause in a wide spectrum of neurological diseases, including inherited optic atrophy, Charcot-Marie-Tooth type 2, Leigh syndrome, progressive myoclonic ataxia and lethal congenital pontocerebellar hypoplasia. SLC25A46 is an outer membrane protein, member of the Solute Carrier 25 (SLC25) family of nuclear genes encoding mitochondrial carriers, with a role in mitochondrial dynamics and cristae maintenance. Here we identified a loss-of-function mutation in the *Slc25a46* gene that causes lethal neuropathology in mice. Mutant mice manifest the main clinical features identified in patients, including ataxia, optic atrophy and cerebellar hypoplasia, which were completely rescued by expression of the human ortholog. Histopathological analysis revealed previously unseen lesions, most notably disrupted cytoarchitecture in the cerebellum and retina and prominent abnormalities in the neuromuscular junction. A distinct lymphoid phenotype was also evident. Our mutant mice provide a valid model for understanding the mechanistic basis of the complex SLC25A46-mediated pathologies, as well as for screening potential therapeutic interventions.

## Author summary

Neurodegenerative diseases encompass a wide range of clinical conditions remaining incurable while the genetic and molecular basis of most of these conditions remains unknown due to their heterogeneity and the lack of animal models. Mutations in nuclear genes encoding mitochondrial proteins have recently emerged as novel causalities in diseases affecting the nervous system. SLC25A46, a novel mitochondrial protein, has recently been identified as a pathogenic target in a wide spectrum of rare genetic neurological diseases, including optic atrophy, Charcot-Marie-Tooth type 2, Leigh syndrome, progressive myoclonic ataxia and lethal congenital pontocerebellar hypoplasia. Following a genetic approach, we identified a novel neurological mouse model caused by a functional mutation in the *Slc25a46* gene. Our SLC25A46 mutant mice constitute the first genetic animal model for this disease and represent an ideal tool for elucidating the underlying molecular mechanisms. The aim of this study was to characterize the complex phenotype displayed by the mutant mice in order to understand the mechanistic basis of the SLC25A46-mediated pathologies.

## Introduction

Mitochondria are highly dynamic organelles with a central role in a plethora of cellular processes, including ATP synthesis through oxidative phosphorylation (OXPHOS), metabolism, apoptosis, and reactive oxygen species generation, with important implications for neurodegenerative diseases. Recent discoveries show that mitochondrial shape and bioenergetics are tightly linked. Mitochondria have a characteristic structure as they are surrounded by two membranes: the inner membrane (IM) and the outer membrane (OM). The IM is further subdivided into the inner boundary membrane (IBM), which is adjacent to the OM and the cristae that protrude into the matrix. IM proteins involved in mitochondrial fusion and protein translocation are preferentially located in the IBM, whereas proteins involved in oxidative phosphorylation are enriched in the cristae membrane. A variety of proteins regulate cristae biogenesis, with optic atrophy 1 (OPA1) and the mitochondrial contact site (MICOS) complex being master regulators in this process [1]. The OM separates the mitochondria from the cytosol, yet it allows the passage of metabolites through the voltage-dependent anion channel (VDAC) and nuclear-encoded proteins through the translocase of the outer membrane (TOM) complex [2,3]. Mitochondria fuse and divide dynamically in response to changes in the cellular environment through family members of dynamin-related GTPases. Dynamin-related protein 1 (Drp1) controls mitochondrial fission while mitofusins (MFN) in the outer membrane and OPA1 in the inner membrane regulate mitochondrial fusion. Interestingly, mutations in MFN2 are associated with Charcot-Marie-Tooth type 2 (CMT2) [4] while mutations in OPA1 are the major cause of dominant optic atrophy [5,6]. Recent findings also demonstrate that OPA1 controls mitochondrial dynamics by sensing changes in nutrient availability through mitochondrial solute carriers [7].

Solute Carrier 25 (SLC25) is a large family of more than 50 nuclear-encoded transporters embedded mainly in the inner mitochondrial membrane that shuttle a variety of substrates participating in numerous metabolic pathways [8]. SLC25 members vary greatly in their size, the nature of transported substrates and the modes of transport, whereas common mechanisms of substrate translocation have been proposed [9,10]. To date, numerous SLC25-related rare metabolic diseases have been identified, caused by mutations in 13 genes encoding SLC25 proteins with various symptomatology, depending on the affected metabolic pathways and their significance in specific tissues [11]. Even though the majority of these disease-related SLC25 members have been biochemically characterized through identification of their substrates and correlated metabolic pathways, there are yet two orphan SLC25 members, SLC25A38 and SLC25A46, causing disorders in humans whose substrates have not been identified [11]. SLC25A46 has recently been identified as a cause in heterogeneous clinical conditions, including optic atrophy, axonal peripheral neuropathy Charcot-Marie-Tooth type 2 (CMT2) [12], an optic atrophy spectrum disorder [13], Leigh syndrome [14], progressive myoclonic ataxia with optic atrophy and neuropathy [15] and lethal congenital pontocerebellar hypoplasia [16]. It has been proposed that human SLC25A46 is a mitochondrial outer membrane protein with a role in mitochondrial dynamics and cristae maintenance that interacts with MFN2, OPA1 and members of the MICOS complex [12,14]. However, the effects of its ablation in animal models *in vivo* have not been addressed yet. Thus, the unavailability of genetic animal models has compromised the in-depth dissection of the related pathology and its correlation with the human disorders.

In the present study, following a forward genetics approach, we identified a loss-of-function mutation in the *Slc25a46* gene that causes lethal neuropathology in mice. Mutant mice manifest the main clinical features described in patients, including ataxia, optic atrophy and cerebellar hypoplasia as well as additional previously unidentified neuropathological alterations, while they were rescued by expression of the human ortholog. The aim of this study was to characterize the complex phenotype displayed by the mutant mice in order to understand the mechanistic basis of the heterogeneous SLC25A46-mediated pathologies.

## Results

### Identification of a lethal recessive neurological phenotype with lymphoid abnormalities in mice through random mutagenesis

**Fig 1:**
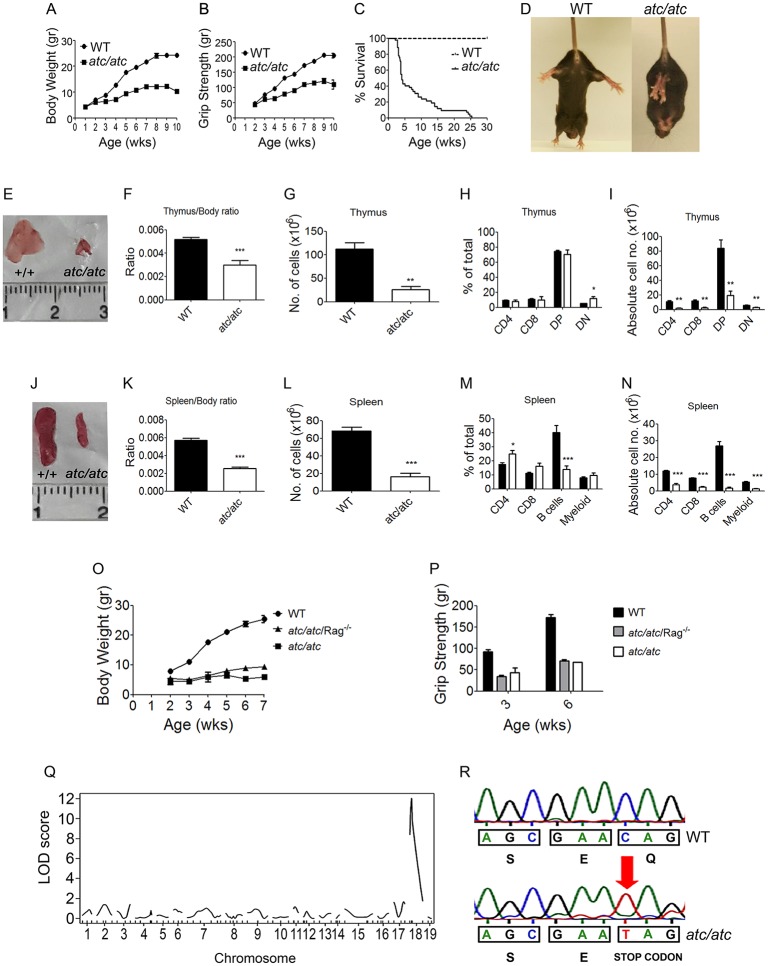
Clinical characterization of the *ataxic* phenotype and identification of the causal mutation in *Slc25a46*. **(A)** Reduced body weight gain (n = 20 per group), **(B)** muscle weakness (n = 20 per group), **(C)** premature lethality (n = 40 per group) and **(D)** loss of hind limb extension reflex during tail suspension in *ataxic* (*atc/atc*) mice as compared to sex matched control (+/+ or *atc*/+) littermates. **(E)** Representative thymi dissected from WT and *atc/atc* mice. **(F)** Reduced thymus/body weight ratios in *atc/atc* mice as compared to WT littermates (n = 17 per group) accompanied by **(G)** lower total thymocyte numbers in *atc/atc* mice (n = 4–5 per group). **(H)** Percentages and **(I)** absolute numbers of thymic subpopulations (CD4+, CD8+, DP: Double Positive CD4+CD8+, DN: Double Negative CD4-CD8-) in WT (black columns, n = 5) and *atc/atc* (white columns, n = 4) littermates as determined by flow cytometry after staining with antibodies against CD4 and CD8. **(J)** Representative spleens dissected from WT and *atc/atc* mice. Reduction in spleen/body weight ratios in **(K)** *atc/atc* mice as compared to WT littermates (n = 17 per group) and **(L)** total splenocyte numbers (n = 5 per group). **(M)** Percentages and **(N)** absolute numbers of splenic subpopulations in WT and *atc/atc* littermates (n = 5 per group) through flow cytometry after staining with antibodies against CD4, CD8, B220 (B cells), CD11b and Gr1 (Myeloid). *Atc/atc* mice were crossed with *Rag2* deficient mice (*Rag*^*-/-*^) that lack mature T and B lymphocytes and **(O)** body weight curves as well as **(P)** grip strength measurements demonstrate that the neurological phenotype is not reversed (WT and *atc/atc*/*Rag*^*-/-*^, n = 5 per group; *atc/atc*, n = 3). Data represent mean values ± SEM. Two-tailed unpaired Student’s *t*-test was performed for statistical analysis. * p≤0.05; ** p≤0.01; *** p≤0.001. **(Q)** Through genome-wide linkage analysis the causal mutation was mapped on chromosome 18. **(R)** Sequencing of genomic DNA samples from WT controls (+/+) and *atc/atc* littermates revealed an exonic C-to-T transition (red arrow) in the *Slc25a46* gene that introduce a premature stop codon.

Following a forward genetics approach through N-ethyl-N-nitrosourea (ENU) random mutagenesis [17,18], we have recently identified a novel mouse model of autosomal recessive neurological phenotype characterized by ataxia, unsteady locomotion, tonic-clonic seizures, limb-clasping during tail suspension, reduced muscle strength, and growth retardation with premature lethality by the age of 3 months (Fig 1A–1D, S1 Video). Because of the prominent neurological symptoms, this phenotype was designated as *ataxic* (*atc*). The first symptoms were noticeable at the second postnatal week with full penetrance in both sexes. From the third week, *atc/atc* mice stopped gaining weight (Fig 1A), their muscle strength began to deteriorate (Fig 1B) and by the fifth week of age the majority of *atc/atc* mice had died (Fig 1C). Heterozygous mice (*atc*/+) were indistinguishable from their wild-type (WT) littermates throughout life. In order to exclude the possibility that *atc/atc* mice succumb to starvation due to difficulties in accessing food caused by weakness and tremor, mashed wet food pellets were added inside the cage. No gross histopathological lesions such as hepatic steatosis were observed in *atc/atc* mice (S1 Fig). Apart from the neurological phenotype, *atc/atc* mice displayed severe thymic and splenic hypoplasia (Fig 1E–1N). Thymi of 4-week-old *atc/atc* mice had approximately 30% of the WT littermate weight and flow cytometric analysis revealed significant reduced cellularity in all four thymic subpopulations, CD4+CD8-, CD4-CD8+, CD4+CD8+, and CD4-CD8- (Fig 1E–1I). *Atc/atc* mice also developed severe splenic hypoplasia (25% of WT weight) with a significant decrease in the absolute number of T lymphocytes (CD4+ and CD8+), B lymphocytes (B220+) and myeloid cells (CD11b+, Gr1+) (Fig 1J–1N). To examine whether the immune system is associated to the neurological phenotype we generated *atc/atc* mice lacking mature T and B lymphocytes upon genomic deletion of *RAG-2* (recombination activating gene) gene that is necessary for immunoglobulin and T-cell receptor gene rearrangements [19]. Clinical evaluation of the double mutant mice showed that the neurological symptoms, survival, body weight, and muscle strength were not reversed (Fig 1O and 1P), implying that the lymphoid and neurological symptoms are distinct.

### Identification of the causal mutation in *Slc25a46* gene

To map the causal mutation we performed genetic linkage analysis using an initial set of 68 Simple Sequence Length Polymorphic markers (SSLPs) in 20 *atc/atc* and 20 not affected F2 mice, and identified clear linkage with chromosome 18 with a LOD score of 12 (Fig 1Q). Fine mapping by analyzing 37 additional F2 mice and screening with 2 Single Nucleotide Polymorphisms (SNPs) located in between the two extreme SSLPs, narrowed down the mutant locus to a genetic region of 8.84 Mbp between D18Mit22 and rs30151517 (S1 Table). Selected candidate gene sequencing, identified a C to T transition at the coding region of the *Slc25a46* gene, introducing a premature termination codon and resulting in a truncated protein of 95 amino acids instead of the 418 amino acids composing WT SLC25A46 (Fig 1R). SLC25A46 is a member of the Solute Carrier 25 (SLC25) family of nuclear genes encoding mitochondrial carriers that shuttle a variety of substrates across the inner mitochondrial membrane [8]. SLC25 members are characterized by the presence of three tandem repeats of about 100 amino acids, each containing two transmembrane alpha helices linked by a large loop. As the primary sequence of the SLC25A46 protein indicates the presence of six conserved transmembrane regions spanning a region between amino acids 100 to 418 [14], the identified nonsense mutation presumably leads to a loss-of-function allele. Functional point mutations in the *SLC25A46* gene have recently been reported in heterogeneous neurological disorders [12,13,14,15,16]. Our mutant mice emerge as the first genetic mouse model for understanding the complex pathophysiological role of the SLC25A46 protein *in vivo*.

### Mouse SLC25A46 is an outer mitochondrial membrane protein with prominent expression in the nervous system

**Fig 2:**
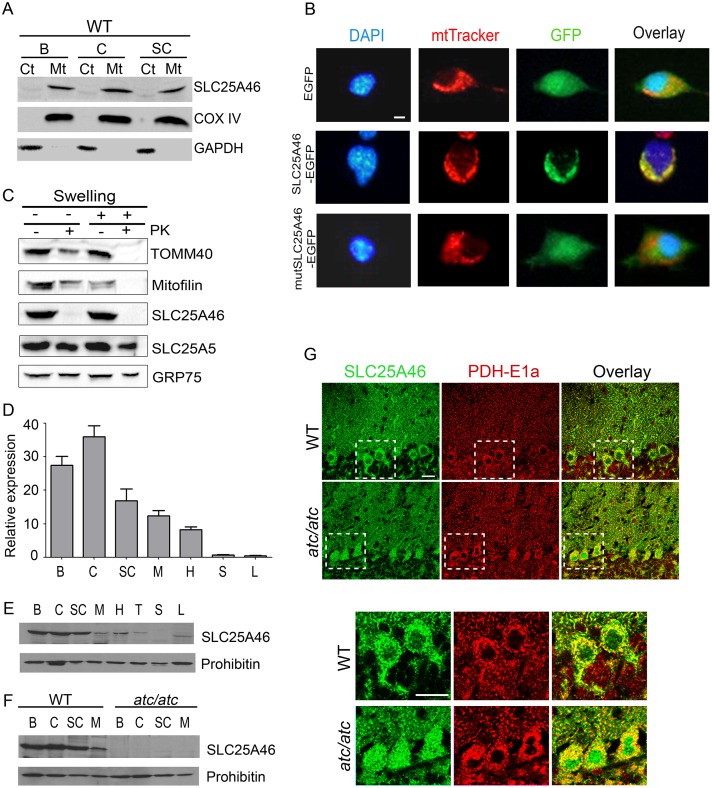
Mitochondrial localization of the SLC25A46 protein. **(A)** Cytosolic (Ct) and mitochondrial (Mt) fractions from cerebrum (B), cerebellum (C) and spinal cord (SC) tissues isolated from WT mice were analyzed by immunoblotting with antibodies for SLC25A46, the mitochondrial protein COX IV and the cytosolic protein GAPDH. **(B)** Fluorescence microscopy of N2A cells transfected with plasmids expressing either EGFP, SLC25A46-EGFP or mutSLC25A46-EGFP carrying the *atc* mutation. DAPI: nuclear staining, mtTracker: mitotracker for mitochondrial staining. Scale bar: 20μm. **(C)** Mitochondria and mitoplasts prepared by hypotonic swelling (Sw) of mitochondria from WT cerebellum, were treated with proteinase K (PK) and analyzed by immunoblotting with antibodies against SLC25A46, the outer membrane protein TOMM40, the intermembrane space protein Mitofilin, the integral inner membrane protein SLC25A5 and the matrix protein GRP75. Analysis of the SLC25A46 expression pattern in various tissues from WT mice at the mRNA level with **(D)** qPCR for mouse *Slc25a46* and western blots in mitochondrial extracts from **(E)** WT mice and **(F)** WT and *atc/atc* mice with antibodies against the SLC25A46 and the mitochondrial protein prohibitin. Cerebrum (B), cerebellum (C), spinal cord (SC), muscle (M), heart (H), thymus (T), spleen (S) and liver (L). **(G)** Immunofluorescence labeling of 4-week-old mid-sagittal cerebellar sections for SLC25A46 shows a clear co-localization with the mitochondrial marker PDH-E1a in PCs of WT mice while a more diffuse distribution is apparent within the PC somata of *atc/atc* mice indicative of cytoplasmic localization. The insets are shown at higher magnification in the lower panel. Scale bar 20 μm.

The majority of SLC25 members are embedded in the inner mitochondrial membrane. The mitochondrial localization of mouse SLC25A46 was confirmed by immunoblot analysis in WT central nervous system (CNS) tissues (Fig 2A) and by fluorescence microscopy upon expression of a chimeric SLC25A46-EGFP protein in the N2A mouse neuroblastoma cell line (Fig 2B). Similarly to human SLC25A46, the mouse ortholog localizes to the outer mitochondrial membrane, as shown upon swelling and proteinase K protection assays in mitochondria isolated from the cerebellum of WT mice (Fig 2C) or cell lines (S2 Fig). Analysis of SLC25A46 expression profile showed prominent expression in the cerebrum, cerebellum and spinal cord (Fig 2D and 2E), and complete absence of the full-length 46 kD protein in mitochondria from *atc/atc* mice (Fig 2F). However, the mRNA expression levels of *Slc25a46* remained unaffected in *atc/atc* mice (S3 Fig), indicating that the truncating mutation does not lead to nonsense-mediated mRNA decay. According to the "50 nucleotide rule" this premature termination codon is not predicted to subject to decay, as it is located 38 nucleotides apart from the next exon-exon junction [20]. Double immunofluorescence labelling for SLC25A46 and the alpha subunit of the mitochondrial enzyme pyruvate dehydrogenase E1 (PDH-E1a) revealed high expression of SLC25A46 in mitochondria of WT cerebellar Purkinje cells (PCs), while a diffuse cellular distribution was identified in *atc/atc* PC somata (Fig 2G), similarly to N2A cells expressing the mutant SLC25A46-EGFP protein (Fig 2B). These results demonstrate that the mutant SLC25A46 protein is not embedded in the mitochondrial membrane, thus being ensued with a compromised function.

### Functional ablation of SLC25A46 results in cerebellar hypoplasia, compromised Purkinje cell dendritic arborization and reduced synaptic connectivity in *atc/atc* mice

**Fig 3:**
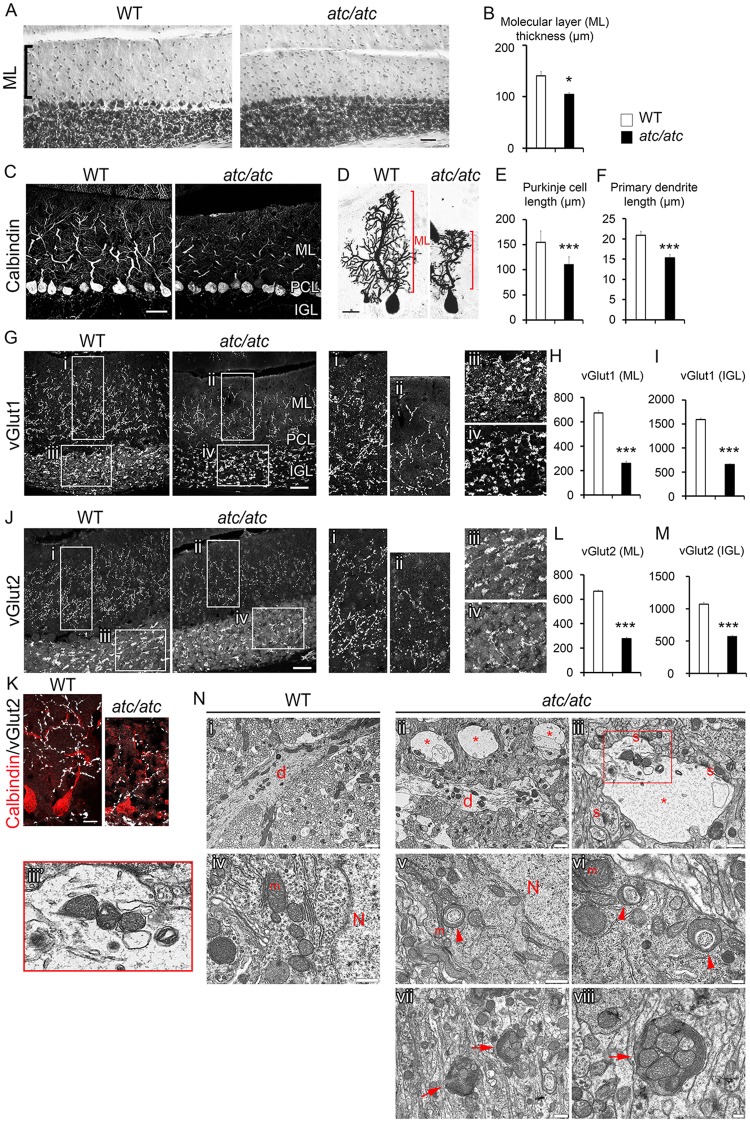
Compromised Purkinje cell dendritic arborization, reduction in glutamatergic synapses and ultrastructural alterations in the cerebellar cortex of *atc/atc* mice. **(A)** Toluidine-blue staining of cerebellar mid-sagittal sections from 4 week-old mice shows reduced thickness of the molecular layer (ML) in *atc/atc* animals. **(B)** Graph showing quantification of average ML thickness (n = 4 per genotype, p = 0.018). **(C)** Calbindin immunostaining and **(D)** Golgi impregnation reveal a significant reduction in Purkinje cell dendritic arborization in *atc/atc* mice. **(E)** Quantification of Purkinje cell dendritic length as marked in red in (D). **(F)** Quantification of the branching distance of the thick primary dendrite into secondary and tertiary dendrites (n = 25 cells pooled from 3 mice per genotype, p = 0.0001). **(G)** Immunostaining of sagittal cerebellar sections from 4 week-old mice reveals a significant decrease in the expression of vGlut1 both in PC-parallel fiber synapses in the ML as well as in granule cell-mossy fiber synapses in the IGL of the cerebellar cortex of *atc/atc* mice. The insets are shown at higher magnification in (i-iv). **(H, I)** Quantification of vGlut1 fluorescence intensity in ML and IGL (n = 4 mice per genotype, p<0.00001). **(J)** Similarly, immunostaining for vGlut2 revealed a significant reduction PC-climbing fiber synapses in the ML and granule cell-mossy fiber synapses in the IGL. The insets are shown at higher magnification in (i-iv). **(K)** The reduction in vGlut2-positive synapses is illustrated at higher magnification in double immunofluorescence micrographs showing calbindin-positive PCs and vGlut2-positive puncta. **(L, M)** Quantification of vGlut2 fluorescence intensity in ML and IGL (n = 4 mice per genotype, p<0.00001). Statistical significance was determined by two-tailed unpaired Student’s *t*-test. Data represent mean values ± SEM Scale bar 40μm in **A, C, G, J**; 20μm in **D, K**. **(N)** Electron microscopy shows **(i)** a longitudinally sectioned dendrite (d) across the field, with normal mitochondria and neurofilament distribution in WT mice; **(ii, iii)** Longitudinal (d) and transverse (*) sections of *atc/atc* PC dendrites, containing disorganized neurofilaments and remnants of mitochondria and other membrane organelles [inset in **(iii’)**], indicating degeneration. Synapses (S) are marked; (iv) Part of WT PC soma close to the nucleus (N) containing mitochondria and other organelles. **(v)** Part of *atc/atc* PC soma close to the nucleus (N) and **(vi)** PC dendrite containing atypical mitochondria (m) with cytoplasmic inclusions (arrowheads). **(vii and viii)** Structures of concentrically arranged, flattened cisternae, surrounding clusters of mitochondria (arrows) in the *atc/atc*. Scale bars (i, ii), 1 μm; (iii, iv, v, vii), 500 nm; (vi, viii), 200 nm. 2 mice per genotype were observed with similar findings.

Because of the prominent SLC25A46 expression in the nervous system of WT mice and the *ataxic* phenotype of mutant animals, we examined the brain for possible histological alterations. Macroscopic examination showed no overt abnormalities in the gross anatomy of *atc/atc* brain. However, histological staining revealed a selective 25% reduction in the size of the cerebellum (S4 Fig) with prominently reduced thickness of the molecular layer (ML) (Fig 3A and 3B). Although PC numbers and alignment were not affected, PC dendrites were severely stunted (Fig 3C–3F). PCs are the main output neurons of the cerebellar cortex receiving signals that modulate their activity through multiple synapses on their elaborate dendritic arbors [21]. Parallel fibers (the axons of granule neurons) extending from the internal granule layer (IGL) of the cerebellar cortex and climbing fibers originating from the inferior olivary nucleus form excitatory glutamatergic synapses on PC dendrites [22]. Additionally, mossy fibers coming from pre-cerebellar nuclei indirectly target PCs, via formation of synapses with granule cell dendrites within the IGL [23]. To elucidate the basis of cerebellar hypoplasia and PC impairment, we examined the distribution of glutamatergic synapses between PCs and parallel or climbing fibers in the ML, as well as the distribution of mossy fiber synapses in the IGL, which indirectly target PCs via granule cells, using antibodies against the vesicular glutamate transporters vGlut1 and vGlut2. Our analysis showed a significant reduction in vGlut1 and vGlut2 immunoreactivity both in the ML and the IGL, indicating a paucity of glutamatergic synapses in *atc/atc* mice (Fig 3G–3M). Interestingly, a reduction in vGlut1 and vGlut2 immunoreactivity was visible at postnatal day 9 when no obvious defects in PC morphology were discernible as yet (S5 Fig). This feature, which is compatible with the ponto-cerebellar hypoplasia observed in the human disease [16], suggests that the stunted PC dendritic growth observed later could be linked to or exacerbated by the compromised glutamatergic input. An alternative hypothesis is that the reduced glutamatergic input is a consequence rather than a cause of the underdeveloped PC dendritic arborization, possibly due to primary impaired mitochondrial dynamics.

To examine the dendritic ultrastructure of PCs we employed electron microscopy (EM) in ultrathin sections of the cerebellum of 4-week-old *atc/atc* and WT mice. Unlike the well-preserved appearance of PC dendrites in the molecular layer of the cerebellum in WT mice (Fig 3Ni), several degenerated PC dendrites were identified in *atc/atc* mice as judged by their disorganized cytoskeleton, often containing remnants of mitochondria and other organelles (Fig 3Nii and 3Niii and inset Fig 3Niii’). Additionally, numerous atypical mitochondria with cytoplasmic inclusions were found both in the soma and dendrites of *atc/atc* PCs (Fig 3Nv and 3Nvi) that were not present in the WT (Fig 3Ni and 3Niv). Structural configurations containing concentric stacks of flattened cisternae that surrounded clusters of mitochondria were frequently observed in *atc/atc* (Fig 3Nvii and 3Nviii) but not in WT mice. Surprisingly, other alterations associated with mitochondrial ultrastructural organization or distribution, were not noted.

Apart from aberrations in cerebellar cytoarchitecture, we observed significant astrogliosis and microgliosis in the spinal cord of mutant mice, indicating high levels of neuroinflammation, as estimated by the increase in GFAP^+^ astrocytes and resting (Iba1^+^/CD68^-^) or activated (Iba1^-^/CD68^+^) microglia by comparison with WT mice (S6 Fig). A reduction in the size of the spinal cord was also evident in *atc/atc* mice without any differences in neuronal density, motor neuron appearance or the profile of cholinergic nerve terminals (S7 Fig).

### *Atc/atc* mice display cellular alterations in the retina and optic nerve

**Fig 4:**
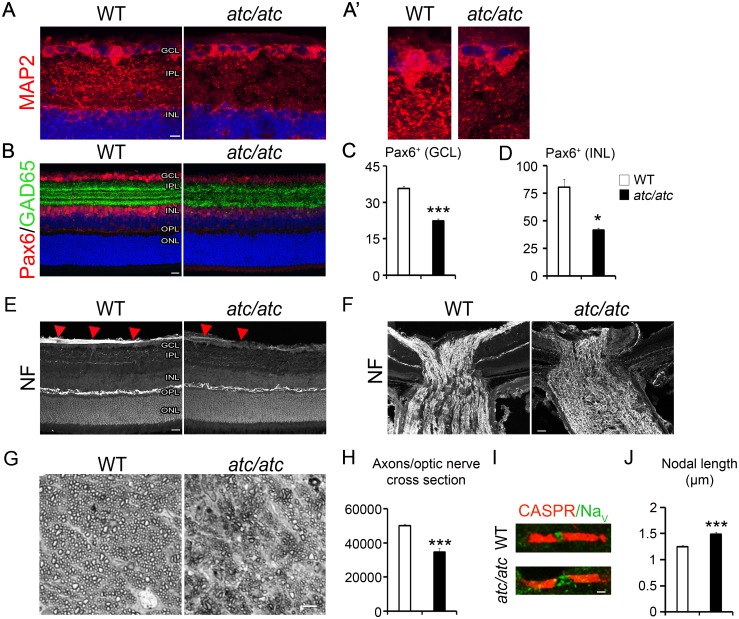
Cellular alterations in the retina and optic nerve of *atc/atc* mice. **(A)** and at higher magnification **(A’)** Confocal images of 4-week-old mouse retinas showing reduced MAP2 immunostaining in the internal plexiform layer (IPL) of *atc/atc* mice where the RGC dendrites lie. **(B)** Amacrine cells immunoreactive for Pax6 and GAD65 are significantly reduced in the *atc/atc* retina. Quantification of Pax6^+^ cells in the ganglion cell layer (GCL) **(C)** and the inner nuclear layer (INL) **(D)** (n = 3 mice per genotype, p = <0.0001 for the GCL and p = 0.03 for the INL). Confocal images of 4-week-old mouse retina **(E)** and optic nerve head **(F)**, showing reduced NF immunoreactivity in the innermost part of the retina where RGC axons lie and disorganization of RGC axons in the optic nerve of *atc/atc* mice. **(G)** Toluidine blue stained semi-thin optic nerve sections of 4-week-old WT and *atc/atc* mice. **(H)** Quantification of axonal numbers per optic nerve section (n = 4 nerves per genotype, p = 0.007). **(I)** Double immunofluorescence of a myelinated fiber in the optic nerve labeled for CASPR and Na_v_. Scale bars (A), 10 μm; (B, E, F) 20 μm; (G) 5μm; (I) 1 μm. **(J)** Quantification of Na_v_+ nodal length (n = 206 WT and 212 *atc/atc* nodes pooled from 3 mice/genotype, p<0.00001; two-tailed unpaired Student’s *t*-test; mean values ± SEM).

As the converging phenotype of SLC25A46-mediated pathologies is optic atrophy, we further investigated whether *atc/atc* mice develop histological optic lesions. Retinal ganglion cells (RGCs) receive visual information from photoreceptors located in the retina, which are transmitted to the brain via their long axons converging to form the optic nerve. Although RGC numbers and alignment were not affected, RGC dendrites were clearly underdeveloped as observed using the somatodendritic marker MAP2 (Fig 4A and 4A’). Additionally the Pax6^+^ amacrine cells in the inner nuclear layer (INL) and the ganglion cell layer (GCL) as well as the GAD65^+^ GABAergic amacrine cells, both forming synapses with RGCs [24], were significantly reduced (Fig 4B–4D). Further, aberrations in RGC axons were noted in the retina with reduced expression of neurofilament (NF) protein and a disorganized appearance within the optic nerve head of mutant mice, indicative of optic atrophy [25] (Fig 4E and 4F). This was confirmed in semi-thin cross-sections of the *atc/atc* optic nerve where morphometric analysis demonstrated a 30% reduction in the number of myelinated axons (Fig 4G and 4H).

Finally, the nodes of Ranvier appeared more elongated in myelinated fibers of the *atc/atc* optic nerve (Fig 4I and 4J) and cerebellum (S4D and S4E Fig), suggesting disorganization of nodal structures and alterations in action potential propagation by saltatory conduction [26,27], in the absence of gross myelination defects (S4A–S4C Fig).

### Improper neuromuscular junction innervations in *atc/atc* mice

**Fig 5:**
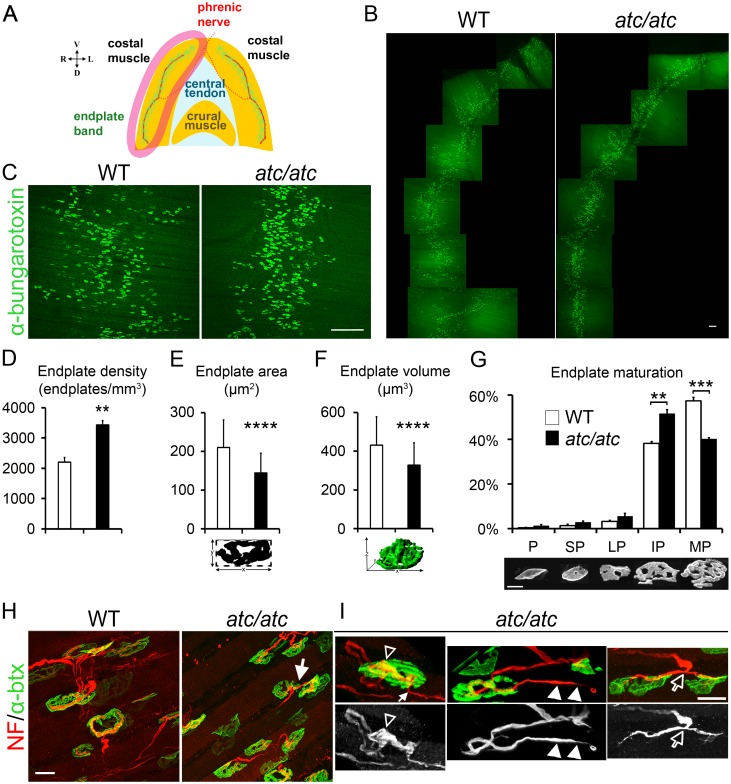
NMJ alterations in *atc/atc* mice. **(A)** Schema of the abdominal view of the mouse diaphragm where the endplate band along each costal muscle is visualized in green and the innervating phrenic nerve is shown in red. (Orientation cross: V, ventral; D, dorsal; R, right; L; left). **(B** and higher magnification in **C)** Confocal images of whole mount diaphragm specimens (costal muscle; see drawing in A) from 4-week old WT and *atc/atc* littermates. The endplates are visualized by binding of FITC conjugated a-btx on AChR clusters (green) and show a narrower distribution along the endplate band in the *atc/atc* diaphragm. **(D)** Quantification of endplate density (n = 3, p = 0.0043). **(E)** Quantification of individual end plate area (n = 204 WT and 347 *atc/atc* endplates pooled from 3 mice/genotype, p<0.00001). **(F)** Quantification of individual endplate volume by Imaris surface module (n = 150 WT and 296 *atc/atc* endplates pooled from 3 mice/genotype, p<0.00001). **(G)** Classification of WT and *atc/atc* endplates based on maturation level (see Materials and methods). P: Plaque; SP: Plaque with small perforations; LP: Plaque with large perforations; IP: Immature pretzel (p = 0.0023); MP: Mature pretzel (p = 0.00005) [n = 4; 200 endplates were characterized per sample), ** p≤0.01; *** p≤0.001; **** p≤0.0001; two-tailed unpaired Student’s *t*-test; mean values ± SEM in (D, G); mean values ± SD in (E, F)]. **(H-I)** Representative images of WT and *atc/atc* diaphragm depicting the increased density and smaller size endplates in *atc/atc* visualized by FITC-a-btx (green), coupled with NF immunostaining [red in merged (J, K) and white in single-channel images (I)]. The arrow in (H) indicates abnormal bifurcation of one fiber to innervate two distinct endplates in *atc/atc*. **(I)** Representative images of abnormal NMJs observed in *atc/atc* mice: poly-innervation of an endplate (white arrow); increased axonal ramification within the endplate (empty arrowheads); axon terminal protrusions extending beyond endplate boundaries reminiscent of retraction bulbs (white arrowheads); an axon that bifurcates and the two fibers innervate two distinct endplates (empty arrows) as also shown in (H). Scale bars (B, C), 200 μm; (G), 10 μm, (H), 20 μm; (I), 10 μm.

Neuromuscular junction alterations were identified in the diaphragm of *atc/atc* mice (Fig 5). A significant increase in the number and density of endplates was detected, resulting in a narrower endplate band along the costal parts of the muscle (Fig 5A–5C). Quantification of the mean endplate area and volume revealed a significantly reduced size of individual endplates in *atc/atc* mice (Fig 5E and 5F). A number of structural and functional alterations occur upon mammalian NMJ development during the first postnatal weeks [28,29]. At birth, NMJs are innervated by multiple motor axonal inputs while excess branches withdraw progressively through the dynamic, activity-dependent process of synapse elimination, so that by 2 weeks after birth the majority of mouse NMJs become innervated by a single motor axon [30]. At the same time, the post-synaptic area transitions from a plaque-like structure to a complex pretzel-shaped domain with multiple-folds and perforations, formed as acetylcholine receptors (AChRs) migrate to closely appose to the pre-synaptic input [31]. Analysis of endplate classification into 5 characteristic stages of maturation according to their shape indicated a shift towards immature pretzel endplates (Fig 5G). Additional abnormal features included endplate poly-innervation, increased axonal ramification within the endplate, axon terminal protrusions extending beyond the endplate boundaries and bifurcation of single axons into two endplates (Fig 5H and 5I). Retarded NMJ maturation and improper innervation are early hallmarks of CMT2D [32] and in a vital muscle such as the diaphragm, these features are indicative of a functional deficit, which could cause or contribute to early lethality [33]. On the other hand, analysis of semi-thin cross sections of the sciatic nerve for myelination (g-ratio), axon diameter and number of myelinated or non-myelinated axons (Remak bundles) did not yield any differences between *atc/atc* and WT mice (S8 Fig). However, fine alterations in the ultrastructure of peripheral nerves cannot be excluded at present evidence.

### Genetic confirmation of the *Slc25a46* mutation

**Fig 6:**
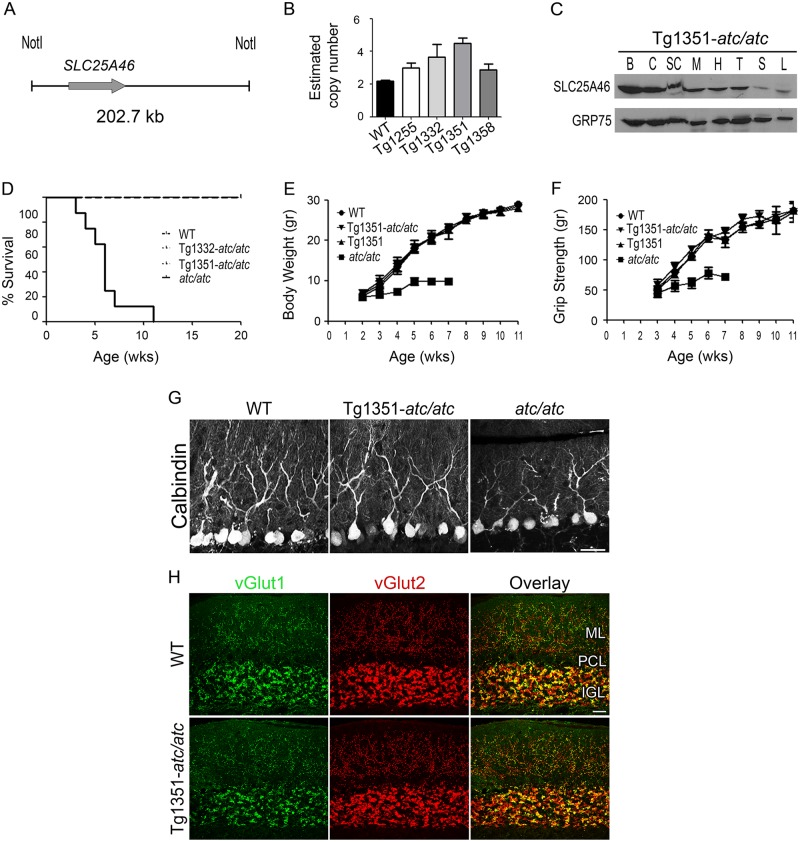
Complete rescue of the *ataxic* phenotype through complementation with the human *SLC25A46* gene. **(A)** Schematic representation of the NotI digested 202.7Kb genomic fragment used for the generation of *TghuSLC25A46* mice. **(B)** Transgene copy number determination by real-time PCR in four transgenic lines, Tg1255, Tg1332, Tg1351 and Tg1358 (n = 6 per group), compared to the two *Slc25a46* copies of the WT mice, using a primer pair common for both mouse and human *Slc25a46* genes. **(C)** Expression pattern of human SLC25A46 on mitochondrial extracts from cerebrum (B), cerebellum (C), spinal cord (SC), muscle (M), heart (H), thymus (T), spleen (S) and liver (L) tissues of *TghuSLC25A46/Slc25a46*^*atc/atc*^ mice (Tg1351-*atc/atc*) through immunoblotting with antibodies against SLC25A46 and the mitochondrial protein GRP75. Complete rescue of **(D)** premature lethality (n = 8 per group), **(E)** body weight gain (n = 6 per group) and **(F)** muscle weakness (n = 6 per group) in *atc/atc* mice expressing human SLC25A46 (Tg1351-*atc/atc*). All mice used were sex matched littermates. **(G)** Confocal images of cerebellar sagittal sections immunostained for calbindin illustrate the restored PC phenotype and ML thickness in transgenic “rescue” Tg1351-*atc/atc* one-year old mice, as compared to WT and *atc/atc* mice. **(H)** Immunofluorescence of vGlut1 and vGlut2 shows normal distribution, in the ML and IGL of the cerebellum of Tg1351-*atc/atc* mice. Scale bar 40 μm.

To confirm that the nonsense mutation identified in the *Slc25a46* gene is causal for the *ataxic* phenotype, genetic rescue experiments were performed by introducing a human *SLC25A46* transgene (*TghuSLC25A46*) into the *Slc25a46*^*atc/atc*^ background (Fig 6, S9 Fig). As the human and the mouse SLC25A46 proteins exhibit 89% identity, they are expected to have common function. To achieve a physiological expression pattern of the human transgene in the mouse, a 202.7 kb genomic fragment containing the whole human *SLC25A46* gene (Fig 6A) was isolated from a Bacterial Artificial Chromosome (BAC) clone and was used for pronuclear microinjections. This BAC fragment did not contain any coding regions of genes other than *SLC25A46*, ensuring exclusive production of human SLC25A46 protein. Four transgenic founders were generated carrying either one copy (Tg1255, Tg1332, and Tg1358) or two copies (Tg1351) of the transgene, as shown by quantitative real-time PCR (qPCR) (Fig 6B), all of which were fertile and appeared healthy, exhibiting no obvious neurological symptoms. From these lines, Tg1332 and Tg1351 (Fig 6B) were selected for crosses with *atc/atc* mice. The expression of the human SLC25A46 protein in the *Slc25a46*^*atc/atc*^ background, which lacks the endogenous full-length SLC25A46 protein, followed a physiologically relevant pattern in various tissues tested, including the cerebrum, cerebellum, spinal cord, muscle, heart, thymus, spleen and liver (Fig 6C, S9A Fig) as compared to the mouse SLC25A46 (Fig 2E). The clinical phenotype of these mice was fully reversed as they had normal life span, normal body weight and grip strength compared to their WT and *Slc25a46*^*atc/atc*^ littermates (Fig 6D–6F, S9B and S9C Fig). To confirm the phenotypic reversal at the cellular level, we performed immunofluorescence labeling on cerebellum mid-sagittal sections of one-year-old mice with antibodies against calbindin, vGlut1 and vGlut2. The profile of calbindin expression in the double mutant mice, *TghuSLC25A46/Slc25a46*^*atc/atc*^, was indistinguishable from that in their WT littermates (Fig 6G). Moreover, the compromised dendritic arborization of PCs in *atc/atc* mice was fully restored as well as the thickness of the molecular layer. The distribution of glutamatergic synapses, as defined by the expression of vGlut1 and vGlut2, also appeared normal in the “rescued” transgenic mice, both in the molecular and the granular layer (Fig 6H). In conclusion, phenotypic analysis of these mice showed complete reversal of the clinical features and the cerebellar histopathological abnormalities displayed by *atc/atc* mice, thus providing genetic confirmation for the causality of the mutation identified in the *Slc25a46* gene.

### Loss of SLC25A46 modifies the levels of putative interactors

Recently, SLC25A46 has been shown to interact with proteins that regulate cristae maintenance, including MFN2, OPA1 and the MICOS complex [14]. To investigate whether *in vivo* ablation of SLC25A46 affects the levels of such proteins in the cerebellum, we followed a comparative proteomic approach using LC-MS/MS and label free quantitation in mitochondrial enriched protein extracts isolated from 4-week-old *atc/atc* mice and WT littermates (Table 1, S2 Table). As expected, SLC25A46 was identified in the CNS of WT mice and was absent in the mitochondrial extracts from *atc/atc* cerebellum. The depletion of SLC25A46 resulted in significantly altered levels of mitochondrial proteins critically involved in fusion and fission, including OPA1, MFN2, and mitochondrial fission factor (MFF). As regards the levels of the MICOS members Mitofilin (IMMT), CHCHD3 and CHCHD6, they were significantly increased in the absence of SLC25A46. Upregulated levels were also noted for the MICOS interacting protein SAMM50, the outer membrane proteins VDAC1 and VDAC2, members of the translocases of the outer (TOMM22, TOMM40 and TOMM70) and the inner (TIMM22 and TIM44) membrane complexes. Collectively, our data indicate that ablation of SLC25A46 affects remarkably the protein levels of mitochondrial proteins involved in mitochondrial dynamics and cristae maintenance in the cerebellum, a tissue mostly affected in *atc/atc* mice.

**Table 1:** Label free proteomic analysis.

Protein name	p-value	Log 2 Fold Change	Change direction	Significance
SLC25A46	Not Applicable	∞ (only in WT)	extinguished	***
**Fusion/Fission**				
OPA1	0.000639	0.81	up	***
MFN2	0.024845	0.43	up	*
MFF	0.000139	1.14	up	***
**MICOS complex**				
IMMT	0.004325	0.65	up	**
CHCHD3	0.006141	0.56	up	**
CHCHD6	0.049151	0.55	up	*
**SAM**				
SAMM50	0.003500	0.46	up	**
**Porins**				
VDAC1	0.012494	0.39	up	*
VDAC2	0.017235	0.27	up	*
**Translocation complex**				
TIMM22	0.000239	1.02	up	***
TIMM44	0.000352	1.02	up	***
TOMM22	0.001122	0.64	up	**
TOMM40	0.002626	0.51	up	**
TOMM70a	0.021190	0.27	up	*

Up-regulated mitochondrial proteins in *atc/atc* mice compared to WT mice. Functional categories and selected proteins, p-value and average log2 fold differences (four biological and three technical replicas) have been calculated as described in Methods. Not Applicable: p-value due to absence of SLC25A46 protein in *atc/atc* mice.

^∞^, the difference is infinite due to lack of detection in one of the compared groups. Up, up-regulated in *atc/atc* mice.

*p<0.05,

**p<0.01, and

***p<0.001.

## Discussion

Following a forward genetics approach through ENU-mediated random mutagenesis, we identified a novel mouse model of severe autosomal recessive neurological disease characterized by ataxia, tonic-clonic seizures, unsteady locomotion, lymphoid abnormalities and growth retardation with early lethality. Through genetic linkage analysis, we identified a nonsense point mutation in the coding region of the *Slc25a46* gene that introduces a premature stop codon and results in a loss-of-function truncated protein. All aspects of this lethal neurological phenotype were rescued through the expression of the human ortholog in transgenic mice, providing a genetic confirmation for the causality of the identified mutation. This is the first mouse model of human SLC25A46-mediated pathologies that have recently been reported by five independent research groups with heterogeneous clinical manifestations including optic atrophy, axonal peripheral neuropathy Charcot-Marie-Tooth type 2 (CMT2) [12], an optic atrophy spectrum disorder [13], Leigh syndrome [14], progressive myoclonic ataxia with optic atrophy and neuropathy [15] and lethal congenital pontocerebellar hypoplasia [16]. Mutant mice manifest the main clinical features identified in patients, including ataxia, optic atrophy and cerebellar hypoplasia while histopathological analysis revealed previously unseen lesions, most notably disrupted cytoarchitecture in the cerebellum and retina, neuroinflammation in the spinal cord, prominent abnormalities in the neuromuscular junction, as well as a distinct lymphoid phenotype.

SLC25A46, a highly conserved protein among various species, is a member of the Solute Carrier 25 (SLC25) family of nuclear genes encoding mitochondrial carriers, characterized by the presence of a tripartite structure, six conserved transmembrane regions and the three-fold repeated signature motif P-X-[DE]-X-X-[RK]. Similar to other SLC25 members with transport activity, the latter motif displays modifications in SLC25A46 making it difficult to predict transport of putative substrates [9,10]. This could be associated with the fact that SLC25A46 is a mitochondrial outer membrane protein both in humans [12,14] and mice according to our results. On the other hand, a role for SLC25A46 in mitochondrial dynamics and cristae maintenance has been recently proposed based on the identification of hyperfused mitochondria with abnormal cristae architecture upon SLC25A46 elimination [12,14]. We could not detect such ultrastructural mitochondrial aberrations in the cerebellar cortex of *atc/atc* mice, although degenerated PC dendrites were clearly identified often containing atypical mitochondria with cytoplasmic inclusions. Nevertheless, it is possible that the recurring presence of mitochondrial clusters surrounded by concentric stacks of flattened cisternae presumably corresponding to endoplasmic reticulum (ER) membranes, may be associated with disturbances in ER-controlled mitochondrial dynamics [14].

The extended N-terminus of SLC25A46 (~ 100 amino acids) could account for recognition functions and interactions with identified partners, including OPA1, MFN2 and members of the MICOS complex [14]. Through its unique features, SLC25A46 seems to be part of a larger interactome. Towards this direction we examined whether *in vivo* loss of SLC25A46 affected the levels of suggested interactors in the cerebellum, a tissue most affected in mutant mice, using a proteomic approach. We demonstrated that loss of SLC25A46 resulted in significantly altered levels of mitochondrial proteins critically involved in fusion and fission including OPA1, MFN2, and mitochondrial fission factor (MFF). Moreover, increased levels were identified for MICOS members, the MICOS interacting protein outer membrane protein SAMM50, VDAC proteins, as well as members of the TOM and TIM complexes. Collectively, our results show that loss of SLC25A46 affects remarkably the mitochondrial interactome in the cerebellum of *atc/atc* mice.

In summary, the *atc/atc* mice displaying the main features identified in patients including ataxia, premature death, cerebellar hypoplasia and optic atrophy constitute a valid model for mechanistic studies and further investigation of the associated human pathology. Towards this scope, formerly unseen histopathological lesions were identified in lymphoid tissues, the cerebellum, retina, spinal cord and NMJ, facilitating the analysis of the complex clinical phenotype caused by SLC25A46 mutations. Further work should elucidate the role of SLC25A46 in mitochondrial metabolism and associated neuropathology.

## Materials and methods

### Mouse husbandry

DBA/2J mice were purchased from the Jackson Laboratories and C57BL/6J mice from the Charles River Laboratories. Mice were maintained and bred under specific pathogen-free conditions in the animal facility of B.S.R.C. (Biomedical Sciences Research Center) ‘Alexander Fleming’. Mice were sacrificed via CO_2_ inhalation followed by cervical dislocation or as otherwise described below.

### Ethics statement

All animal procedures were carried out in strict compliance with the European Directive 2010/63/EU and the Greek National Law 56/2013 for Use of Laboratory Animals, according to the Federation of European Laboratory Animal Science Association recommendations for euthanasia and the National Institutes of Health Guide for Care and Use of Laboratory Animals and in accordance to the Hellenic License for Animal Experimentation at the B.S.R.C. ‘Alexander Fleming’ (Licence Protocol Number 1169/08.03.2012) issued by the Veterinary Department of Athens Prefecture after protocol approval by the Institutional Animal Ethical Committee of B.S.R.C. ‘Alexander Fleming’ (Protocol Number 259/13.02.2012).

### ENU mutagenesis

Mutagenesis and breeding schemes were performed at B.S.R.C. ‘Alexander Fleming’ as previously described [17,34]. Briefly, three weekly doses of 100 mg/kg ENU (Sigma-Aldrich) were administered intraperitoneally into C57BL/6Jx129S6 male mice (G0) which were subsequently crossed to WT C57BL/6Jx129S6 females to produce G1 males. Backcross breeding schemes were set up between G1 males and G2 females to conduct a recessive phenotypic screen for ataxia in G3 progeny. Mutant mice were backcrossed at least for 10 generations on the C57BL/6J inbred strain to obtain congenic mice carrying the *Slc25a46* mutation for phenotypic characterization.

### Grip strength

The Grip Strength Test Meter, Model GS3 (Bioseb) was used. Mice were allowed to stand with four paws on the metal grid and were gently pulled backwards by the tail until they were off the grid. The mean from three repeats was calculated for every measurement.

### Flow cytometry

Thymi and spleens were ground on size-40 metallic mesh discs (Sigma-Aldrich) and cell suspensions were isolated after filtering through 100-μm sheet. Thymocytes were resuspended in PBS and counted using a hematocytometer. Splenocytes were resuspended in Gey’s solution, filtered, pelleted and counted. 3x10^6^ thymocytes were plated in 96 V-Bottom well plates (Costar) and stained with antibodies against CD4-Alexa 700, CD8-Alexa 647, CD44-FITC, CD25-PE (Biolegend, U.S.). 10^6^ splenocytes were plated and stained with antibodies against CD4-Alexa 700, CD8-Alexa 647, B220-PerCP, CD11b-PE and Gr1-FITC (Biolegend, U.S.). BD FACSCanto II Flow Cytometer was used for running the samples and results were analyzed with DIVA software.

### Oil Red O staining

Liver parts were isolated from WT and *atc/atc* mice, mounted on O.C.T. compound (WVR) and snap frozen in liquid N_2_. The samples were let at -80°C O/N. Sections of 10 μm thickness were collected using Leica CM1950 cryotome and processed for staining according to Roy Ellis Staining Protocol. Images were acquired using a Nikon Eclipse fluorescence microscope on 20x magnification. A WT mouse was subjected to 24h starvation as a control of lipid accumulation [35].

### Mapping and sequencing

Heterozygous *+/atc* mice were outcrossed to DBA/2J mice to generate F1 mice that were further intercrossed and produced F2 progeny used in genetic linkage analysis. Genomic DNA samples from F2 mice, both *ataxic* and not affected, were screened for Simple Sequence Length Polymorphisms (SSLPs) on 4% high resolution agarose gels (Sigma-Aldrich) and Single Nucleotide Polymorphisms (SNPs) by pyrosequencing (Pyromark ID, Biotage AB). Results were statistically analyzed using the qtl library of R (The R Foundation for Statistical Computing, version 3.2.2) as previously described [36]. For sequencing purposes, PCR products were extracted from agarose gel and sent for analysis at MWG Biotech AG.

For *Slc25a46* genotyping the following primers were used for pyrosequencing on tail DNA: F, 5’-CTC CCT GTT CCA TAT ATA TCA GAA TTT G-3’; R, 5’-AGA TTA CCT TGC AAG TCC AAT ACC-3’ and sequencing primer 5’-TCT TAT TCC TTT TAT TTT AG-3’.

### Isolation of tissue mitochondria

Tissue mitochondria isolation was performed via differential centrifugation. Mitochondria Isolation Buffer (MIB) (210 mM mannitol, 70 mM sucrose, 5 mM HEPES, 1 mM EGTA, 0.5% BSA, pH 7.2) supplemented with proteinase inhibitors cocktail (Roche) was used to homogenize approximately 100 mg of tissue in a 1 ml glass mortar and pestle, manually, on ice. The homogenate was centrifuged (1000 g, 5 min, 4°C), the supernatant was kept on ice and the pellet was resuspended in MIB and centrifuged again (1000 g, 5 min, 4°C). The two supernatants were mixed and centrifuged (12000 g, 10 min, 4°C) twice to acquire the mitochondrial pellet and the cytoplasmic fraction. The mitochondrial pellet was washed with 500 μl MIB and after centrifugation was resuspended in a minimal volume of MIB. Protein concentration was determined using the Bradford method (Bio-Rad Protein Assay).

### Immunoblotting

After subcellular fractionation, 20 mg of mitochondria were mixed with denaturating buffer (10% SDS, 50% Glycerol, 60 mM Tris/HCl pH 6.8, 100 mM DTT, 0.1%, Bromophenol blue, 5% 2-Mercaptoethanol) and separated by SDS-PAGE (12% gel, 0.1% SDS) at 120 V for 3 h at room temperature. Proteins were transferred to 0.45-μm nitrocellulose membrane (Santa Cruz Biotechnology) performing Wet Electroblotting (Tank Transfer, Bio-Rad) at 110 V for 1 h, on ice. The membranes were blocked with 5% BSA (Sigma-Aldrich) in PBS/Tween-20 (for SLC25A46, Translocase of Outer Mitochondrial Membrane 40/TOMM40, SLC25A5) or 5% skimmed milk in TBS (for Cytochrome c Oxidase Subunit IV/COX IV, Glyceraldehyde 3-Phosphate Dehydrogenase/GAPDH, Mitofilin, Glucose Regulated Protein/GRP75, Prohibitin) for 2 h at room temperature. Membranes were then incubated with antibodies against SLC25A46 (goat polyclonal; 1:2500 in 5% BSA in PBS/Tween-20; Santa Cruz Biotechnology), COX IV (rabbit polyclonal; 1:4000 in 5% skimmed milk in TBS; Novus Biologicals), GAPDH (mouse polyclonal; 1:4000 in 5% skimmed milk in TBS; Santa Cruz Biotechnology), TOMM40 (rabbit polyclonal; 1:500 in 5% BSA in PBS/Tween-20; Proteintech), Mitofilin(rabbit polyclonal; 1:500 in 5% skimmed milk in TBS; Proteintech), SLC25A5 (rabbit polyclonal; 1:500 in 5% BSA in PBS/Tween-20; Proteintech), GRP75 (rabbit polyclonal; 1:2000 in 5% skimmed milk in TBS; Santa Cruz Biotechnology) or Prohibitin (rabbit polyclonal; 1:600 in 5% skimmed milk in TBS; NeoMarkers), overnight at 4°C. Membranes were washed with PBS/Tween-20 or TBS and incubated with the HRP-conjugated secondary antibody according to primary antibody’s host species for 1 h at room temperature. Membranes were then washed with PBS/Tween-20 or TBS and protein bands were visualized using the Molecular Imager ChemiDoc XRS+ system (Bio-Rad). Results were analyzed through the Image Lab software (Bio-Rad).

### Fluorescence microscopy on N2A cells

Neuro2A (N2A) cells grown on glass coverslips were transiently transfected using the calcium phosphate (CaCl_2_) method [37] either with a control pEGFP (Clonetech) plasmid, or SLC25A46-pEGFP plasmids containing mouse *Slc25a46* cDNAs, WT or mutant, fused to the C-terminal of the EGFP cDNA. 48 h post-transfection, cells were stained with 150 nM MitoTracker Red (Molecular Probes) in cell culture media for 20 min at 37°C. Samples were washed with PBS/methanol 1:1, fixed in 4% PFA for 20 min at room temperature and permeabilized with methanol for 10 min. Pictures were taken with a Nikon Eclipse fluorescence microscope and analyzed using ImageJ software.

### Swelling and proteinase K protection assay

50 mg of mitochondria were freshly isolated from WT cerebellum and resuspended either in isotonic buffer (250 mM sucrose, 1 mM EDTA, 10 mM Tris/HCl pH 7.6) or hypotonic buffer (1 mM EDTA, 10 mM Tris/HCl pH 7.6) on ice, as previously described [18]. After 45 min incubation, mitochondria were pelleted by centrifugation (12.000 g, 5 min, 4°C). Samples were treated with 50 mg/ml proteinase K (PK, +) for 30 min on ice and proteinase K was inactivated by the addition of 2 mM PMSF. Mitochondria were pelleted by centrifugation and all samples were analyzed through immunoblotting.

### Reverse transcription and quantitative expression analysis

Total RNA was extracted from various tissues by the guanidinium thiocyanate phenol-chloroform method according to the manufacturer’s instructions using Tri-Reagent (MRC Inc.). Concentration and quality of RNA was determined with a NanoDrop ND-1000 spectrophotometer. DNase I (Sigma-Aldrich) treatment, for removal of residual DNA, was followed by cDNA synthesis using 2 mg of total RNA, 0.5 mg of oligo d(T) 18 primer (New England Biolabs) and M-MLV reverse transcriptase (Sigma-Aldrich). qPCR was performed at 58°C with a primer pair for *Slc25a46* gene (see ‘Transgenic copy number determination’) and a primer pair for B2M gene F, 5’-TTC TGG TGC TTG TCT CAC TGA-3’ and R, 5’-CAG TAT GTT CGG CTT CCC ATT C-3’ used as normalization control. Standard curves were generated for both genes, CT values were determined and data were analyzed by the comparative CT method (Bio-Rad RelQuant). For each experiment, two technical and three biological replicas were used.

### Generation and screening of transgenic mice

Human BAC clone RP11-671K7 (ImaGenes, GmbH) was digested with NotI and products were analyzed by Pulsed Field Electrophoresis. A 202.693 bp fragment containing the human *Slc25a46* gene was isolated upon electrophoresis in a 4% low melting agarose gel and digestion with β-agarase (New England Biolabs). This fragment was microinjected into fertilized (C57BL/6J x CBA/J) F2 oocytes as previously described [18,38,39]. Microinjections and embryo implantations were carried out by Transgenics & Gene Targeting Facility at B.S.R.C. 'Alexander Fleming'. Screening of transgenic mice was performed by PCR analysis on genomic DNA with primers that hybridize a sequence of the human but not the mouse gene as follows: F, 5'-AAT CAC GTG CTC CGA AGA CT-3'; R, 5'-TAA CCC CTC ATC CCT GTG TC-3'.

### Transgene copy number determination

Determination of transgene copy number was performed by quantitative real-time PCR (qPCR) using tail DNA from *TghuSlc25a46* mice and WT littermates. For the reaction SsoFast EVA Green Supermix (Bio-Rad) and a pair of primers specific for both mouse and human *Slc25a46* gene were used: F, 5'-TCT GAC GTT ATA CTT TAC CC-3'; R, 5'-CAG TCT CTC ATT CCC TCA TA-3'. The nuclear gene *HuR* was used as normalization control for each sample: F, 5’-AGG ACA CAG CTT GGG CTA CG-3’ and R, 5’-CGT TCA GTG TGC TGA TTG CT-3’. Standard curves were generated for both primer pairs and CT values were determined and analyzed by the comparative CT method. The values of both *Slc25a46* and *HuR* genes were extrapolated from their respective standard curves for each transgenic line. qPCR was performed using on a Rotor-Gene 6000 RT-PCR machine (Corbett Life Science).

### Tissue processing and immunofluorescence

Mice 4-weeks old or at postnatal day 9 (P9), were sacrificed by exposure to isoflurane and transcardially perfused with fixative (4% paraformaldehyde in phosphate-buffered saline). Brain, eye bulbs with attached the optic nerve, spinal cord and dorsal root ganglia were dissected out and post-fixed overnight at 4°C in the same fixative. Tissues were cryoprotected in 30% w/v sucrose solution in PBS, for 2 days at 4°C, embedded in O.C.T. compound (VWR Chemicals) and frozen at -80°C. A series of 4–5 sagittal sections, 20-μm thick, through the midline of the cerebellar hemispheres were collected and stored at -20°C until processed for immunohistochemistry. Similarly, eye bulbs with the optic nerve were cut in sagittal sections at the level of the optic nerve head. Spinal cords were cryosectioned transversely at the lower thoracic/lumbar level. The diaphragm, after dissection, was separated into the two costal muscle parts that were further processed as whole mount preparations, as previously described [40]. Cryosections were thawed and subjected to antigen retrieval in 10 mM sodium citrate solution, pH 6, followed by 1 h blocking of non-specific sites with 5% normal donkey serum (NDS), simultaneously with permeabilization using 0.2% v/v Triton X-100 in PBS. Primary antibodies diluted in 2.5% NDS in PBS were applied overnight at 4°C, followed by incubation with appropriate secondary antibodies for 2 h at room temperature. Primary antibodies used were: goat polyclonal anti-SLC25A46 (N-13) (1:50; Santa Cruz Biotechnology); rabbit polyclonal anti-Calbindin (1:1000; Chemicon); mouse monoclonal anti-pyruvate dehydrogenase E1 alpha subunit (PDHE1a; 1:50; Santa Cruz Biotechnology); mouse monoclonal anti-vesicular glutamate transporter 1 (vGlut1; 1:500; Millipore); guinea pig polyclonal anti-vesicular glutamate transporter 2 (vGlut2; 1:10000; Millipore); mouse monoclonal anti-beta III tubulin (TUJ1; 1/200; Covance); rabbit polyclonal anti- 2΄,3-cyclic-nucleotide 3΄-phosphodiesterase (CNPase; 1:200; Cell Signaling); rabbit polyclonal anti-contactin associated protein (CASPR 1:2000; gift from Prof. D. Karagogeos at the Institute of Molecular Biology and Biotechnology, Crete, Greece); mouse monoclonal anti-voltage gated sodium channels (Na_v_clone K58/35; 1:500; Sigma-Aldrich); mouse-monoclonal anti-neurofilament (NF; 1:100; Sigma-Aldrich) rabbit polyclonal anti-glial fibrillary acidic protein (GFAP; 1:500; Dako); rabbit polyclonal anti-ionized calcium-binding adapter molecule 1 (Iba-1; 1:400, Cell Signaling); rat anti-CD68 (1:100; Serotec); mouse anti-Neuronal Nuclei (NeuN; 1:500; Chemicon-Millipore); goat anti-choline acetyl-transferase (ChAT; 1:300; Millipore, gift from Dr Zagoraiou); goat anti-vesicular acetylcholine transporter (vAchT; 1:2000; Millipore, gift from Dr Zagoraiou); mouse monoclonal anti-microtubule-associated protein 2 (MAP2; 1:200; Millipore); rabbit polyclonal anti-glutamic acid decarboxylase 65 (GAD65; 1:100; Cell Signaling); mouse monoclonal anti-Pax6 (Pax6; 1:100; Developmental Studies Hybridoma Bank). Secondary antibodies (all from Molecular Probes) used for immunofluorescence were conjugated to Alexa Fluor 488 or 546 and cell nuclei were counterstained with TO-PRO-3 (1:1000; Molecular Probes). FITC-conjugated alpha-bungatotoxin (a-btx; 1:200; Molecular Probes) binding on acetylcholine receptors (AChRs) was used to label the endplates on diaphragm samples. Prolong Gold antifade curing mountant (Molecular Probes) was used for mounting. Images were acquired using Leica TCS SP and Leica TCS-SP5II confocal microscopes.

### Toluidine blue staining of cerebellar sections and measurements

Cryosections were thawed, washed with phosphate-buffered saline (PBS) for 5 min, and then transferred in Toluidine blue solution (0.5% w/v in PBS) (Sigma-Aldrich) for 20 sec. Excessive staining was washed off in PBS for as long as necessary, sections were dehydrated through successive dilutions of ethanol (from 70% to 100%), followed by 2 x 10 min washes with Histolene clearing reagent (CellPath). Samples were mounted with DPX mounting medium (Sigma-Aldrich) and observed under a Zeiss-Axiophot up-right light microscope. The average thickness of ML and average cerebellum area from 5 mice per genotype, were calculated.

### Golgi impregnation

For Golgi staining [41], cerebella of adult mice (4-week-old) were dissected out and fixed in 3.7% PFA/1% methanol in PBS overnight at 4°C. Tissue was then incubated for 3 days in 3% potassium bichromate in the dark at room temperature with daily changes. Cerebella were transferred in 2% silver nitrate, incubated overnight at room temperature in the dark and cut in 50-μm sagittal sections using a vibrating microtome (Leica VT1000E). Sections were air-dried and mounted in DPX mounting medium (Sigma-Aldrich). The length of each Purkinje cell (distance from opposite end of the cell soma to the tip of the longest dendrite) was measured for at least 20 cells in a mid-sagittal section, for 3 mice per genotype in digital images obtained under a Zeiss-Axiophot up-right light microscope using a 63x objective.

### Image analysis

#### Fluorescence intensity

The distribution of glutamatergic synapses between PCs and parallel or climbing fibers in the ML, as well as the distribution of mossy fiber synapses in the IGL, was examined using antibodies against the vesicular glutamate transporters vGlut1 and vGlut2. For evaluation of the expression levels of vGlut1 and vGlut2, fluorescence intensity was measured as pixel intensity on single channel stacks of confocal images, acquired under the same settings, as previously described [42,43]. The laser power was set to the lowest level that would be adequate for high signal/noise ratio acquisition and would not bleach the specimen. Gain and offset were set to constant levels optimized so as to avoid over- and under- exposure acquisition. Each series in the confocal stack was averaged 3 times and the step size was 0.5 μm while image resolution was 1024 x 1024 pixels. Image processing for quantification of fluorescence intensity was performed using the ImageJ software, after selecting the region of interest within the internal granule layer (IGL) and the molecular layer (ML), on single channel stacks using the free-hand selection tool and setting the threshold at a value that was kept constant throughout. Measurements were performed on each single image of the confocal stack and then added up and normalized to the corresponding area. Analysis was performed on postnatal day 9 and 4-week-old cerebella, and 3 animals were analyzed per genotype and per age tested.

#### Pax6 counts in retina

On confocal images of sagittal retinal sections immunostained for Pax6, fields of the same size (110503 sq. microns) were sampled and the number of Pax6^+^ cells was counted in ImageJ, separately for the GCL and INL. Values from 3 fields were averaged per mouse (n = 3 mice per genotype).

#### Nodal length

For estimation of the nodal length of myelinated fibers in the optic nerve and in the white matter of the cerebellum, images from double immunostained samples for the voltage-gated sodium channels Na_v_ that cluster in this region to facilitate saltatory conduction and contactin-associated protein (CASPR) that marks the paranodes, were acquired by sequential scanning under the confocal microscope using 63x objective and 3x zoom, with the step-size set to 0.5 μm and 1024 x 1024 pixels resolution. In the ImageJ software the length of the Na_v_ positive area flanked by CASPR positive paranodes was quantified. Values were pooled from 3 mice per genotype for each tissue.

#### Diaphragm analysis

Analysis of the number, density, area, volume and maturity of endplates was performed as follows. Endplate density was estimated on confocal images of FITC-a-btx labeled endplates that covered the total area of the endplate band on each costal part of the muscle, acquired using 10x objective and 2x zoom. Using the surface module of Imaris software (v.8.2.0) the endplates were recognized as individual objects and their number was divided by the corresponding tissue volume calculated as (image area) x (image depth) for each image and added up for the total number of images per sample. To estimate the mean size of endplates, confocal images of individual endplates were acquired using 63x objective and 2x zoom and (a) the maximum projection area of each endplate was calculated using the ImageJ software after thresholding the a-btx positive area to obtain a binary image as that shown below the corresponding graph in Fig 3g; (b) the volume of each endplate was calculated using the surface module of Imaris software by obtaining 3D images as shown below the corresponding graph in Fig 3h. For both (a) and (b) values were obtained from 150–350 endplates pooled from 3 mice per genotype, as specified in the figure legend. For quantification of their maturation level the endplates were categorized into 5 distinct developmental stages and the stage frequency patterns of the two genotypes were compared [40]. Images below the corresponding graph in Fig 3i are representative of each endplate maturation stage, classified as follows. P: Plaque; Endplates appearing as an oval or round plaque of contiguous staining for AChRs. SP: Plaque with a single or multiple small perforations with a diameter smaller than 1/5th of the endplate diameter. LP: Plaque with a single or multiple large perforations with a diameter greater than 1/5th of the endplate diameter. Single perforations might reach the outer border of the endplate, but strip-like structures typical of pretzels, are not found. IP: Immature pretzel, in which strip-like structures appear. The strips of the nascent pretzels exhibit a varying width. MP: Mature pretzel, in which the strips of the pretzel exhibit a homogeneous width. Approximately 200 endplates were characterized per sample (n = 4).

#### Estimation of astrogliosis in the spinal cord

On confocal images of 3 transverse sections per animal at the lower thoracic/lumbar spinal cord immunostained for GFAP, 4 fields of the same size (29675 sq. microns) per section were randomly selected in respective areas of the gray matter and the number of GFAP^+^ cells counted in ImageJ was expressed as the percentage of total TO-PRO-3 counterstained nuclei. Values from 12 fields were averaged per mouse (n = 3 mice per genotype).

#### Estimation of microgliosis in the spinal cord

On confocal images of 3 transverse sections per animal at the lower thoracic/lumbar spinal cord double immunostained for Iba1 which marks both resting and activated microglia/macrophages and CD68 which marks activated microglia/macrophages, the number of Iba1^+^ cells and the number of activated Iba1^+^/CD68^+^ cells (also exhibiting phagocytic morphology) was counted per total section area calculated in ImageJ using the free-hand selection tool. Values from 3 sections were averaged per mouse (n = 3 mice per genotype).

#### Estimation of the population of NeuN^+^ neurons

On confocal images of 3 transverse sections per animal at the lower thoracic/lumbar spinal cord immunostained for NeuN which marks all neuronal cells, the total number of positive cells was counted and normalized per total section area in Image J. Values from 3 sections were averaged per mouse (n = 3 mice per genotype).

### Electron microscopy and morphometry

For conventional electron microscopy, mice were perfused with 2.5% glutaraldehyde—4% paraformaldehyde in 0.1 M phosphate buffer, pH 7.4. Cerebella, optic nerves and sciatic nerves were removed and fixed overnight at 4°C in the same fixative. Tissues were dissected into 1-mm cubes and post-fixed with 1% osmium tetroxide for 1h at 4°C. They were then passed through a graded series of ethanol, followed by propylene oxide (PO), infiltrated gradually in a mixture of Epon/Araldite resins diluted in PO and then embedded in fresh epoxy resin mixture. Finally, the specimens were allowed to polymerize at 60°C for 24h. Semithin sections (1-μm thick) were stained with toluidine blue. Ultrathin sections were cut with a Diatome diamond knife at a thickness of 65nm on a Leica EM UC7 ultramicrotome (Leica Microsystems, Vienna, Austria), were mounted onto 300 mesh copper grids and stained with uranyl acetate and lead citrate. Sections were examined with a Philips 420 transmission electron microscope at an acceleration voltage of 40 kV and images were acquired with a Megaview G2 CCD camera (Olympus SIS, Münster, Germany).

#### Morphometry

Toluidine blue-stained 1-μm semi-thin cross sections of optic and sciatic nerves were observed under an upright Zeiss Axiophot microscope and images were captured using a Leica DC300 camera. For the optic nerve, the numbers of myelinated axons were counted per field in four randomly sampled fields per section, using ImageJ, and the total number of fibers was calculated for the total area of the cross section. For the sciatic nerve, the total numbers of myelinated axons or Remak-bundles of non-myelinated axons were counted per cross-section, using ImageJ. The diameters of myelinated axons and axons plus myelin were calculated by hand-tracing and measuring the corresponding circumferences, using ImageJ. The g-ratio as an index of myelination, was calculated for each fiber by dividing the inner diameter of the axon to the total diameter of the myelinated fiber. A minimum of 100 axons were measured in each nerve and values were averaged from 3–4 nerves per genotype.

### Proteomic analysis

#### Protein sample preparation

Crude mitochondrial fraction was isolated as described above.

#### Lysis of mitochondria

Purified mitochondria were resuspended in 150 μl lysis buffer containing 100 mM Tris-HCl, pH 7.6, 4% SDS and freshly made 100 mM DTT. Samples were incubated for 3 min at 95°C, followed by 20 minute incubation in a sonication water bath in order to shear the DNA. Finally, the samples were centrifuged at 17000xg for 30 min at 4°C and the supernatants were transferred to new tubes.

#### Protein digestion

The protein extracts were processed according to the Filter Aided Sample Preparation (FASP) protocol [44] using spin filter devices with 10kDa cutoff (Sartorius, VN01H02). 150 μl lysate were diluted in 8 M Urea/100 mM Tris-HCl pH 8.5, the filters were extensively washed with the urea solution, treated with 10 mg/ml iodoacetamide in the urea solution and incubated for 30 min in the dark for the alkylation of cysteines. The proteins on the top of the filters were washed three times with 50 mM ammonium bicarbonate and finally the proteins were digested adding 1 μg trypsin/LysC mix in 80 μl 50 mM ammonium bicarbonate solution (Mass spec grade, Promega) and incubated over night at 37°C. The peptides were eluted by centrifugation, followed by speed-vac-assisted solvent removal, reconstitution in 0.1% formic acid, 2% acetonitrile in water and transferring to LC-MS glass sample vials. Peptide concentration was determined by nanodrop absorbance measurement at 280 nm.

#### Ultra high pressure nanoLC

2.5 μg peptides were pre-concentrated with a flow of 3 μl/min for 10 min using a C18 trap column (Acclaim PepMap100, 100μmx2cm, Thermo Scientific) and then loaded onto a 50 cm C18 column (75 μm ID, particle size 2 μm, 100Å, Acclaim PepMap RSLC, Thermo Scientific). The binary pumps of the HPLC (RSLCnano, Thermo Scientific) consisted of solution A (2% v/v ACN in 0.1% v/v formic acid) and solution B (80% ACN in 0.1% formic acid). The peptides were separated using a linear gradient of 4% to 40% B in 450 min at a flow rate of 300 nl/min. The column was placed in an oven operating at 35°C.

#### LC-MS/MS

The purified peptides were analyzed by HPLC MS/MS coupled to an LTQ Orbitrap XL Mass spectrometer (Thermo Fisher Scientific, Waltham, MA, USA) equipped with a nanospray source. Full scan MS spectra were acquired in the orbitrap (*m*/*z* 300–1600) in profile mode and data-dependent acquisition with the resolution set to 60,000 at *m*/*z* 400 and automatic gain control target at 10^6^ ions. The six most intense ions were sequentially isolated for collision-induced (CID) MS/MS fragmentation and detection in the linear ion trap. Dynamic exclusion was set to 1 min and activated for 90 sec. Ions with single charge states were excluded. Lockmass of m/z 445.120025 was used for internal calibration. Xcalibur (Thermo Scientific) was used to control the system and acquire the raw files.

#### Data analysis

The raw files were analyzed using MaxQuant (version 1.5.3.30) [45,46] using the complete Uniprot proteome of *Mus musculus (version of 7/3/2016)* and a common contaminants database by the Andromeda search engine. Search parameters were strict trypsin specificity, allowing up to two missed cleavages. Oxidation of methionines and N-terminal acetylation were set as variable modifications. Cysteine carbamidomethylation was used as a fixed modification. The protein and peptide false discovery rate (FDR) was set to 1% for both proteins and peptides with a minimum length of seven amino acids that was determined by searching a reverse database. Protein abundance was calculated on the basis of the normalized spectral protein intensity as label free quantitation (LFQ intensity) enabling the “match between runs” option (set at 0.7 min). LFQ was performed with a minimum ratio count of 2. The statistical analysis was performed using Perseus (version 1.5.3.2). Proteins identified as contaminants, “reverse” and “only identified by site” were filtered out. The LFQ intensities were transformed to logarithmic (log2(x)). The replicas (four biological and three technical) were grouped together. The protein groups were filtered to obtain at least eight valid values across the twelve replicates and filtered so at least two peptides were present per valid value. The zero intensities were imputed—replaced by normal distribution, assuming that the corresponding protein has low abundance in the sample (imputation criteria: width 0.1 and down shift 1.8). A total of 2216 label free quantified proteins were subjected to statistical analysis and GO analysis, in which 525 were mitochondrial (based on GO cellular component). Two-sided Student’s *t*-test was performed for the comparison of the WT versus *atc/atc* mouse mitochondrial proteomes using p-value for truncation (p-value threshold of 0.05).

### Statistical analysis

Two tailed unpaired Student’s *t*-test was performed using Prism 5 (GraphPad Software). p-value were symbolized as follows: *p<0.05; **p<0.01; ***p<0.001; ****p<0.0001.

## Supporting information

S1 Fig *Atc/atc* mice do not develop hepatic steatosis. Oil Red O staining of 4-week old male liver cryosections. **(A)** 24h starvation in WT mice (control) leads to accumulation of triglycerides in liver (steatosis) as lack of phosphatidylcholine synthesis limits the export of excess triglyceride from liver in lipoproteins, **(B)** WT and **(C)** *atc/atc* mice with *ad libitum* food access. Neutral triglycerides and lipids were stained with Oil Red O (red). The nuclei were stained with haematoxylin (purple). Scale bar: 150 μm.
(TIF)

S2 Fig Swelling and proteinase K protection assay on isolated mitochondria of (A) N2A cells and (B) HEK293T cells. 50 mg of mitochondria were incubated in isotonic buffer (Sw, -) or hypotonic buffer (Sw, +) in the absence (PK, -) or presence (PK, +) of proteinase K. Samples were subsequently analyzed by immunoblotting with antibodies against SLC25A46, the outer membrane protein TOMM40, the intermembrane space protein Mitofilin, the integral inner membrane protein SLC25A5 and the matrix protein GRP75.
(TIF)

S3 Fig Normal mRNA production in *atc/atc* mice. qPCR analysis in Brain (Cerebrum), Cerebellum and Spinal Cord from WT and *atc/atc* mice. Specific primers designed to hybridize to the 8^th^ exon of *Slc25a46* gene were used to confirm the absence of mRNA decay in *atc/atc* mice.
(TIF)

S4 Fig Cerebellum of *atc/atc* mice: Reduced size, normal appearance of gross myelination, but elongated nodes of Ranvier in the myelinated fibers. **(A,C)** Confocal images of sagittal cerebellar sections immunostained for the neuronal marker beta-3 tubulin (TUJ1, red) and the myelin protein CNPase (green). Nuclei are counterstained with TO-PRO-3 (blue). **(B)** Quantification of mid-sagittal cerebellum area (n = 4, p = 0,0029). Scale bar (a), 400 μm; (c), 40 μm. **(D)** Double immunostaining for the paranodal marker CASPR and voltage-gated sodium channels (Na_v_) clustered at the nodes of Ranvier in myelinated fibers of the cerebellar cortex. Scale bar = 2 μm. **(E)** Quantification of the nodal length based on Na_v_-channel immunostaining in *atc/atc* mice and WT littermates (n = 485 WT and 587 *atc/atc* nodes pooled from 3 mice per genotype, p<0.00001). Statistical significance was determined by two-tailed unpaired Student’s *t*-test. Data represent mean values ± SEM.
(TIF)

S5 Fig Cellular alterations in the cerebellum of P9 mice. **(A)** Immunofluorescence labeling of P9 cerebellar mid-sagittal sections with SLC25A46, reveals diffused expression in the calbindin-positive PC somata of *atc/atc* mice. No differences were noted in the PC dendritic tree at this age. **(B)** Immunostaining of sagittal cerebellar sections reveals a significant decrease in the expression of vGlut1, both in the ML and the IGL of *atc/atc* mice. The insets are shown at higher power magnification. **(C)** Quantification of fluorescence intensity in the ML (p = 0.0028) and **(D)** in the IGL (p = 0.06) (n = 3 mice per genotype). **(E)** Immunostaining of sagittal cerebellar sections shows significant reduction in the expression of vGlut2, both in the ML and the IGL of *atc/atc* mice. **(F)** Quantification of fluorescence intensity in ML (p = 0.078) and **(G)** in the IGL (p = 0.0008) (n = 3 mice per genotype). Statistical significance was determined by two-tailed unpaired Student’s *t*-test. Data represent mean values ± SEM Scale bars, 40 μm.
(TIF)

S6 Fig Astrogliosis and microgliosis indicate neuroinflammation in the spinal cord of the 4 week-old *atc/atc* mouse. Confocal immunofluorescence images of transverse sections of the lower thoracic/lumbar spinal cord of WT and *atc/atc* littermates. **(A)** GFAP labeling (white) at low and the insets at high power micrographs. **(B)** Quantification of the percentage of GFAP^+^ cells showing a statistically significant increase in *atc/atc* (n = 3 mice per genotype, p = 0.019). **(C)** Confocal images of double immunofluorescence against CD68 (activated microglia/macrophages; green in merged images) and Iba-1 (total microglia/macrophages; red in merged images) in spinal cord sections of WT and *atc/atc* mice, at low and the insets at high power magnification. Scale bars, 100 μm and 50 μm for low and high power images, respectively. TO-PRO-3 (blue) shows nuclear counterstaining. **(D)** Quantification revealed a significant increase in the density of Iba1^+^/CD68^-^ cells/ mm^2^ (resting microglia/macrophages, p = 0.002) and Iba1^+^/CD68^+^ cells/ mm^2^ (activated microglia/macrophages, p = 0.0284) in *atc/atc* mice as compared to the WT (n = 3 mice per genotype). * p≤0.05; ** p≤0.01; *** p≤0.001. Statistical significance was determined by two-tailed unpaired Student’s *t*-test. Data represent mean values ± SEM.
(TIF)

S7 Fig Immunohistological analysis of the *atc/atc* mouse spinal cord. **(A)** Toluidine blue staining of spinal cord transverse sections at the lower thoracic/lumbar level; **(B)** Quantification of the area of equivalent, toluidine blue stained spinal cord sections verified the smaller size of the spinal cord as expected due to the reduced growth of *atc/atc* mice as compared to the WT (n = 3 mice per genotype, p = 0.041). Scale bars, 100 μm for A and C (low magnification); 40 μm for E and F. * p≤0.05. Statistical significance was determined by two-tailed unpaired Student’s *t*-test. Data represent mean values ± SEM Confocal immunofluorescence images of transverse sections of the lower thoracic/lumbar spinal cord of WT and *atc/atc* littermates. **(C)** NeuN labeling of neuronal cells (white) and TO-PRO-3 counter stain of nuclei (blue). **(D)** Quantification of the number of NeuN^+^ neurons per area of the spinal cord section (n = 3 mice per genotype, p = 0.415). **(E)** Confocal images of immunofluorescence against ChAT which labels the motor neurons that reside in the ventral horns of the spinal cord. **(F)** Double immunofluorescence against vAchT (white) and NeuN (blue) visualizes the cholinergic synapses on the motor neurons of the spinal cord ventral horns.
(TIF)

S8 Fig Morphometric analysis of the sciatic nerve. **(A)** Representative toluidine blue stained semithin cross sections of 4-week-old WT and *atc/atc* mice. Scale bar: 5 μm. **(B)** Scatter plot of g-ratio as a function of axon diameter indicating a similar distribution of values among WT and *atc/atc* fibers (n = 689 myelinated WT fibers and n = 905 myelinated *atc/atc* fibers pooled from 3 nerves per genotype). **(C)** Histogram representing mean values ± SEM of calculated g-ratios (n = 3 nerves per genotype; p = 0.621). Quantification of **(D)** the mean diameter of myelinated axons (n = 3 nerves per genotype; p = 0.711), **(E)** the number of myelinated axons per nerve (n = 3 nerves per genotype; p = 0.582) and **(F)** the number of Remak bundles of non-myelinated small diameter axons (n = 3 nerves per genotype; p = 0.908). Statistical significance was determined by two-tailed unpaired Student’s *t*-test. Data represent mean values ± SEM.
(TIF)

S9 Fig Complete rescue of the *atc/atc* phenotype through expression of the human *SLC25A46* gene in the transgenic line Tg1332. **(A)** Expression pattern of human SLC25A46 on mitochondrial extracts from cerebrum (B), cerebellum (C), spinal cord (SC), muscle (M), heart (H), thymus (T), spleen (S) and liver (L) tissues of *TghuSLC25A46/Slc25a46*^*atc/atc*^ (Tg1332-*atc/atc*) mice through immunoblotting with antibodies against SLC25A46 and the mitochondrial protein GRP75. Complete rescue of **(B)** body weight gain and **(C)** muscle weakness in *atc/atc* mice expressing human SLC25A46 (Tg1332-*atc/atc*). All mice used were sex matched littermates(n = 6 per group).
(TIF)

S1 Table Fine mapping results indicate the causal mutation between D18Mit22 and rs30151517. SSLPs and SNPs are shown in the left column and respective chromosome 18 position (in bp) are shown in the right column. B, H and D denote homozygosity for mutagenesis strain, heterozygosity and homozygosity for the mapping strain, respectively. Bottom row shows numbers of individual mice. Red and black font denotes *ataxic* and not affected mice, respectively. All *ataxic* mice screened were found homozygous for markers D18Mit225, D18Mit60 and D18Mit158. Four *ataxic* mice were found heterozygous for D18Mit22 and one mouse was found heterozygous for rs30151517 thus defining the chromosomal position where the mutant locus lies. The two candidate region defining markers and their respective positions are shown in bold.
(DOC)

S2 Table Proteomic LFQ intensity and statistical analysis of selected proteins. (XLSX)

S1 Video Neurological phenotype of *atc/atc* mice. (MP4)

## References

[1] Cogliati S, Enriquez JA, Scorrano L (2016) Mitochondrial Cristae: Where Beauty Meets Functionality. Trends Biochem Sci 41: 261–273. 10.1016/j.tibs.2016.01.001 26857402

[2] Colombini M, Blachly-Dyson E, Forte M (1996) VDAC, a channel in the outer mitochondrial membrane. Ion Channels 4: 169–202. 8744209 10.1007/978-1-4899-1775-1_5

[3] Pfanner N, Wiedemann N (2002) Mitochondrial protein import: two membranes, three translocases. Curr Opin Cell Biol 14: 400–411. 12383789 10.1016/s0955-0674(02)00355-1

[4] Zuchner S, Mersiyanova IV, Muglia M, Bissar-Tadmouri N, Rochelle J, et al (2004) Mutations in the mitochondrial GTPase mitofusin 2 cause Charcot-Marie-Tooth neuropathy type 2A. Nat Genet 36: 449–451. 10.1038/ng1341 15064763

[5] Alexander C, Votruba M, Pesch UE, Thiselton DL, Mayer S, et al (2000) OPA1, encoding a dynamin-related GTPase, is mutated in autosomal dominant optic atrophy linked to chromosome 3q28. Nat Genet 26: 211–215. 10.1038/79944 11017080

[6] Delettre C, Lenaers G, Griffoin JM, Gigarel N, Lorenzo C, et al (2000) Nuclear gene OPA1, encoding a mitochondrial dynamin-related protein, is mutated in dominant optic atrophy. Nat Genet 26: 207–210. 10.1038/79936 11017079

[7] Germain M (2015) OPA1 and mitochondrial solute carriers in bioenergetic metabolism. Mol Cell Oncol 2: e982378 10.4161/23723556.2014.982378 27308447 PMC4904881

[8] Palmieri F (2013) The mitochondrial transporter family SLC25: identification, properties and physiopathology. Mol Aspects Med 34: 465–484. 10.1016/j.mam.2012.05.005 23266187

[9] Robinson AJ, Kunji ER (2006) Mitochondrial carriers in the cytoplasmic state have a common substrate binding site. Proc Natl Acad Sci U S A 103: 2617–2622. 10.1073/pnas.0509994103 16469842 PMC1413793

[10] Kunji ER, Robinson AJ (2006) The conserved substrate binding site of mitochondrial carriers. Biochim Biophys Acta 1757: 1237–1248. 10.1016/j.bbabio.2006.03.021 16759636

[11] Palmieri F, Monne M (2016) Discoveries, metabolic roles and diseases of mitochondrial carriers: A review. Biochim Biophys Acta. 10.1016/j.bbamcr.2016.03.007 26968366

[12] Abrams AJ, Hufnagel RB, Rebelo A, Zanna C, Patel N, et al (2015) Mutations in SLC25A46, encoding a UGO1-like protein, cause an optic atrophy spectrum disorder. Nat Genet 47: 926–932. 10.1038/ng.3354 26168012 PMC4520737

[13] Nguyen M, Boesten I, Hellebrekers D, Mulder-den Hartog NM, de Coo I, et al (2016) Novel pathogenic SLC25A46 splice-site mutation causes an optic atrophy spectrum disorder. Clin Genet. 10.1111/cge.12774 26951855

[14] Janer A, Prudent J, Paupe V, Fahiminiya S, Majewski J, et al (2016) SLC25A46 is required for mitochondrial lipid homeostasis and cristae maintenance and is responsible for Leigh syndrome. EMBO Mol Med. 10.15252/emmm.201506159 PMC5009808 27390132

[15] Charlesworth G, Balint B, Mencacci NE, Carr L, Wood NW, et al (2016) SLC25A46 mutations underlie progressive myoclonic ataxia with optic atrophy and neuropathy. Mov Disord. 10.1002/mds.26716 27430653

[16] Wan J, Steffen J, Yourshaw M, Mamsa H, Andersen E, et al (2016) Loss of function of SLC25A46 causes lethal congenital pontocerebellar hypoplasia. Brain. 10.1093/brain/aww212 PMC5840878 27543974

[17] Douni E, Rinotas V, Makrinou E, Zwerina J, Penninger JM, et al (2012) A RANKL G278R mutation causing osteopetrosis identifies a functional amino acid essential for trimer assembly in RANKL and TNF. Hum Mol Genet 21: 784–798. 10.1093/hmg/ddr510 22068587

[18] Ioakeimidis F, Ott C, Kozjak-Pavlovic V, Violitzi F, Rinotas V, et al (2014) A splicing mutation in the novel mitochondrial protein DNAJC11 causes motor neuron pathology associated with cristae disorganization, and lymphoid abnormalities in mice. PLoS One 9: e104237 10.1371/journal.pone.0104237 25111180 PMC4128653

[19] Shinkai Y, Rathbun G, Lam KP, Oltz EM, Stewart V, et al (1992) RAG-2-deficient mice lack mature lymphocytes owing to inability to initiate V(D)J rearrangement. Cell 68: 855–867. 1547487 10.1016/0092-8674(92)90029-c

[20] Neu-Yilik G, Kulozik AE (2008) NMD: multitasking between mRNA surveillance and modulation of gene expression. Adv Genet 62: 185–243. 10.1016/S0065-2660(08)00604-4 19010255

[21] Goldowitz D, Hamre K (1998) The cells and molecules that make a cerebellum. Trends Neurosci 21: 375–382. 9735945 10.1016/s0166-2236(98)01313-7

[22] Gebre SA, Reeber SL, Sillitoe RV (2012) Parasagittal compartmentation of cerebellar mossy fibers as revealed by the patterned expression of vesicular glutamate transporters VGLUT1 and VGLUT2. Brain Struct Funct 217: 165–180. 10.1007/s00429-011-0339-4 21814870

[23] Apps R, Garwicz M (2005) Anatomical and physiological foundations of cerebellar information processing. Nat Rev Neurosci 6: 297–311. 10.1038/nrn1646 15803161

[24] Feng L, Xie X, Joshi PS, Yang Z, Shibasaki K, et al (2006) Requirement for Bhlhb5 in the specification of amacrine and cone bipolar subtypes in mouse retina. Development 133: 4815–4825. 10.1242/dev.02664 17092954 PMC2992969

[25] Bouaita A, Augustin S, Lechauve C, Cwerman-Thibault H, Benit P, et al (2012) Downregulation of apoptosis-inducing factor in Harlequin mice induces progressive and severe optic atrophy which is durably prevented by AAV2-AIF1 gene therapy. Brain 135: 35–52. 10.1093/brain/awr290 22120150

[26] Poliak S, Peles E (2003) The local differentiation of myelinated axons at nodes of Ranvier. Nat Rev Neurosci 4: 968–980. 10.1038/nrn1253 14682359

[27] Arancibia-Carcamo IL, Attwell D (2014) The node of Ranvier in CNS pathology. Acta Neuropathol 128: 161–175. 10.1007/s00401-014-1305-z 24913350 PMC4102831

[28] Shi L, Fu AK, Ip NY (2012) Molecular mechanisms underlying maturation and maintenance of the vertebrate neuromuscular junction. Trends Neurosci 35: 441–453. 10.1016/j.tins.2012.04.005 22633140

[29] Sanes JR, Lichtman JW (1999) Development of the vertebrate neuromuscular junction. Annu Rev Neurosci 22: 389–442. 10.1146/annurev.neuro.22.1.389 10202544

[30] Tapia JC, Wylie JD, Kasthuri N, Hayworth KJ, Schalek R, et al (2012) Pervasive synaptic branch removal in the mammalian neuromuscular system at birth. Neuron 74: 816–829. 10.1016/j.neuron.2012.04.017 22681687

[31] Marques MJ, Conchello JA, Lichtman JW (2000) From plaque to pretzel: fold formation and acetylcholine receptor loss at the developing neuromuscular junction. J Neurosci 20: 3663–3675. 10804208 10.1523/JNEUROSCI.20-10-03663.2000 PMC6772702

[32] Sleigh JN, Grice SJ, Burgess RW, Talbot K, Cader MZ (2014) Neuromuscular junction maturation defects precede impaired lower motor neuron connectivity in Charcot-Marie-Tooth type 2D mice. Hum Mol Genet 23: 2639–2650. 10.1093/hmg/ddt659 24368416 PMC3990164

[33] Osanai S, Akiba Y, Nakano H, Matsumoto H, Yahara O, et al (1992) Charcot-Marie-Tooth disease with diaphragmatic weakness. Intern Med 31: 1267–1270. 1295621 10.2169/internalmedicine.31.1267

[34] Hrabe de Angelis MH, Flaswinkel H, Fuchs H, Rathkolb B, Soewarto D, et al (2000) Genome-wide, large-scale production of mutant mice by ENU mutagenesis. Nat Genet 25: 444–447. 10.1038/78146 10932192

[35] Zeisel SH (2006) Choline: critical role during fetal development and dietary requirements in adults. Annu Rev Nutr 26: 229–250. 10.1146/annurev.nutr.26.061505.111156 16848706 PMC2441939

[36] Broman KW, Wu H, Sen S, Churchill GA (2003) R/qtl: QTL mapping in experimental crosses. Bioinformatics 19: 889–890. 12724300 10.1093/bioinformatics/btg112

[37] Chen C, Okayama H (1987) High-efficiency transformation of mammalian cells by plasmid DNA. Mol Cell Biol 7: 2745–2752. 3670292 10.1128/mcb.7.8.2745-2752.1987 PMC367891

[38] Rinotas V, Niti A, Dacquin R, Bonnet N, Stolina M, et al (2014) Novel genetic models of osteoporosis by overexpression of human RANKL in transgenic mice. J Bone Miner Res 29: 1158–1169. 10.1002/jbmr.2112 24127173

[39] Douni E, Alexiou M, Kollias G (2004) Genetic engineering in the mouse: tuning TNF/TNFR expression. Methods Mol Med 98: 137–170. 10.1385/1-59259-771-8:137 15064438

[40] Bolliger MF, Zurlinden A, Luscher D, Butikofer L, Shakhova O, et al (2010) Specific proteolytic cleavage of agrin regulates maturation of the neuromuscular junction. J Cell Sci 123: 3944–3955. 10.1242/jcs.072090 20980386

[41] Sergaki MC, Guillemot F, Matsas R (2010) Impaired cerebellar development and deficits in motor coordination in mice lacking the neuronal protein BM88/Cend1. Mol Cell Neurosci 44: 15–29. 10.1016/j.mcn.2010.01.011 20153830

[42] Papastefanaki F, Jakovcevski I, Poulia N, Djogo N, Schulz F, et al (2015) Intraspinal Delivery of Polyethylene Glycol-coated Gold Nanoparticles Promotes Functional Recovery After Spinal Cord Injury. Mol Ther 23: 993–1002. 10.1038/mt.2015.50 25807288 PMC4817765

[43] Koutsoudaki PN, Papastefanaki F, Stamatakis A, Kouroupi G, Xingi E, et al (2016) Neural stem/progenitor cells differentiate into oligodendrocytes, reduce inflammation, and ameliorate learning deficits after transplantation in a mouse model of traumatic brain injury. Glia 64: 763–779. 10.1002/glia.22959 26712314

[44] Wisniewski JR, Zougman A, Nagaraj N, Mann M (2009) Universal sample preparation method for proteome analysis. Nat Methods 6: 359–362. 10.1038/nmeth.1322 19377485

[45] Cox J, Mann M (2008) MaxQuant enables high peptide identification rates, individualized p.p.b.-range mass accuracies and proteome-wide protein quantification. Nat Biotechnol 26: 1367–1372. 10.1038/nbt.1511 19029910

[46] Cox J, Hein MY, Luber CA, Paron I, Nagaraj N, et al (2014) Accurate proteome-wide label-free quantification by delayed normalization and maximal peptide ratio extraction, termed MaxLFQ. Mol Cell Proteomics 13: 2513–2526. 10.1074/mcp.M113.031591 24942700 PMC4159666

