# Supplementary figures and images for: Novel insights into SLC25A46-related pathologies in a genetic mouse model
Source: PLoS Genet. 2017 Apr 4;13(4):e1006656. doi: 10.1371/journal.pgen.1006656 (PMC5380310; doi:10.1371/journal.pgen.1006656)

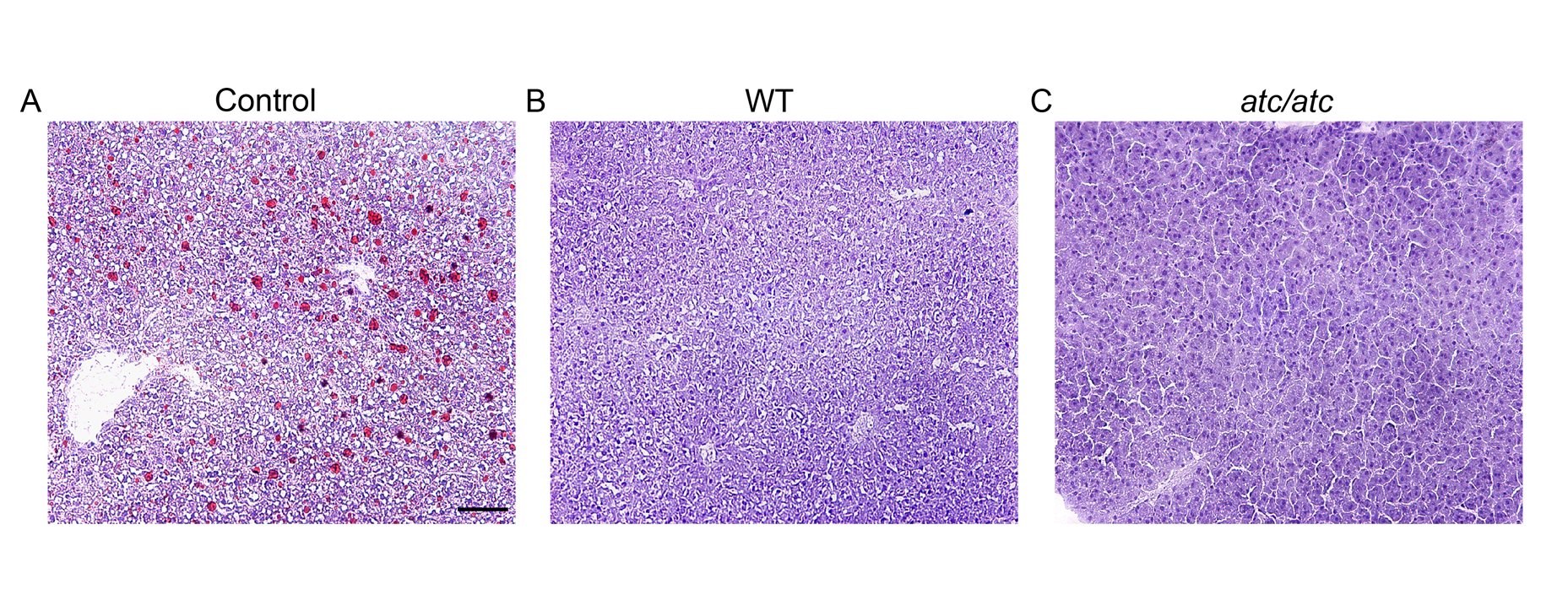

Supplement: S1 Fig — Oil Red O staining of 4-week old male liver cryosections. (A) 24h starvation in WT mice (control) leads to accumulation of triglycerides in liver (steatosis) as lack of phosphatidylcholine synthesis limits the export of excess triglyceride from liver in lipoproteins, (B) WT and (C) atc/atc mice with ad libitum food access. Neutral triglycerides and lipids were stained with Oil Red O (red). The nuclei were stained with haematoxylin (purple). Scale bar: 150 μm. (TIF) [file pgen.1006656.s001.tif]

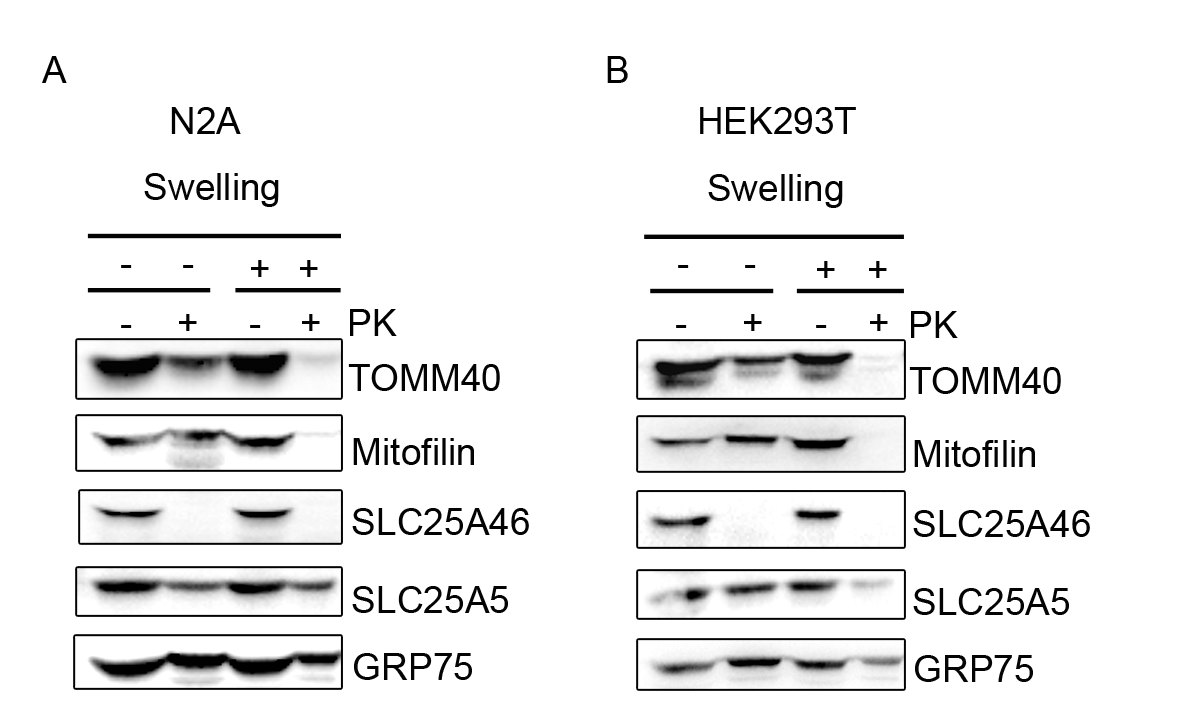

Supplement: S2 Fig — 50 mg of mitochondria were incubated in isotonic buffer (Sw, -) or hypotonic buffer (Sw, +) in the absence (PK, -) or presence (PK, +) of proteinase K. Samples were subsequently analyzed by immunoblotting with antibodies against SLC25A46, the outer membrane protein TOMM40, the intermembrane space protein Mitofilin, the integral inner membrane protein SLC25A5 and the matrix protein GRP75. (TIF) [file pgen.1006656.s002.tif]

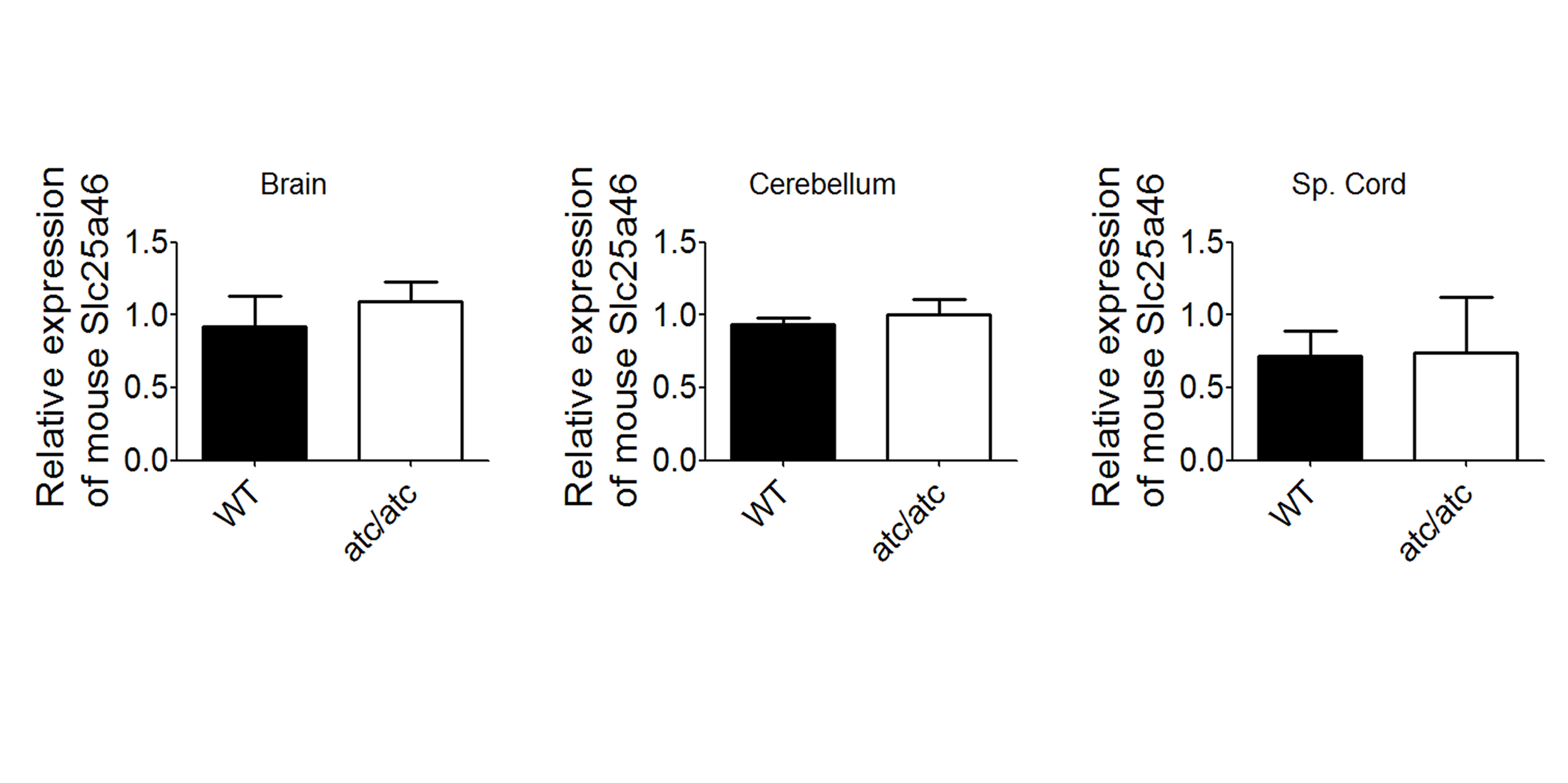

Supplement: S3 Fig — qPCR analysis in Brain (Cerebrum), Cerebellum and Spinal Cord from WT and atc/atc mice. Specific primers designed to hybridize to the 8th exon of Slc25a46 gene were used to confirm the absence of mRNA decay in atc/atc mice. (TIF) [file pgen.1006656.s003.tif]

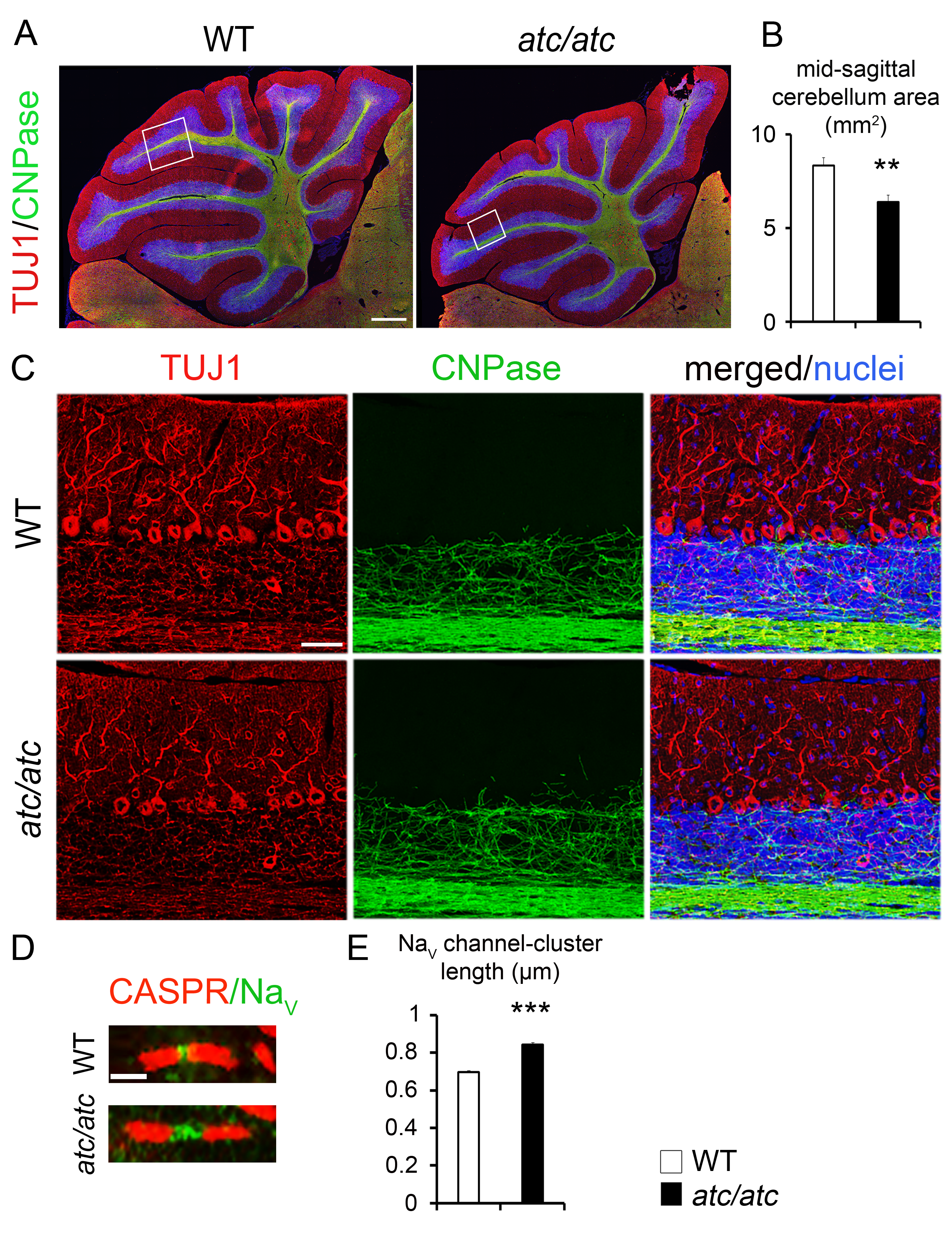

Supplement: S4 Fig — (A,C) Confocal images of sagittal cerebellar sections immunostained for the neuronal marker beta-3 tubulin (TUJ1, red) and the myelin protein CNPase (green). Nuclei are counterstained with TO-PRO-3 (blue). (B) Quantification of mid-sagittal cerebellum area (n = 4, p = 0,0029). Scale bar (a), 400 μm; (c), 40 μm. (D) Double immunostaining for the paranodal marker CASPR and voltage-gated sodium channels (Nav) clustered at the nodes of Ranvier in myelinated fibers of the cerebellar cortex. Scale bar = 2 μm. (E) Quantification of the nodal length based on Nav-channel immunostaining in atc/atc mice and WT littermates (n = 485 WT and 587 atc/atc nodes pooled from 3 mice per genotype, p<0.00001). Statistical significance was determined by two-tailed unpaired Student’s t-test. Data represent mean values ± SEM. (TIF) [file pgen.1006656.s004.tif]

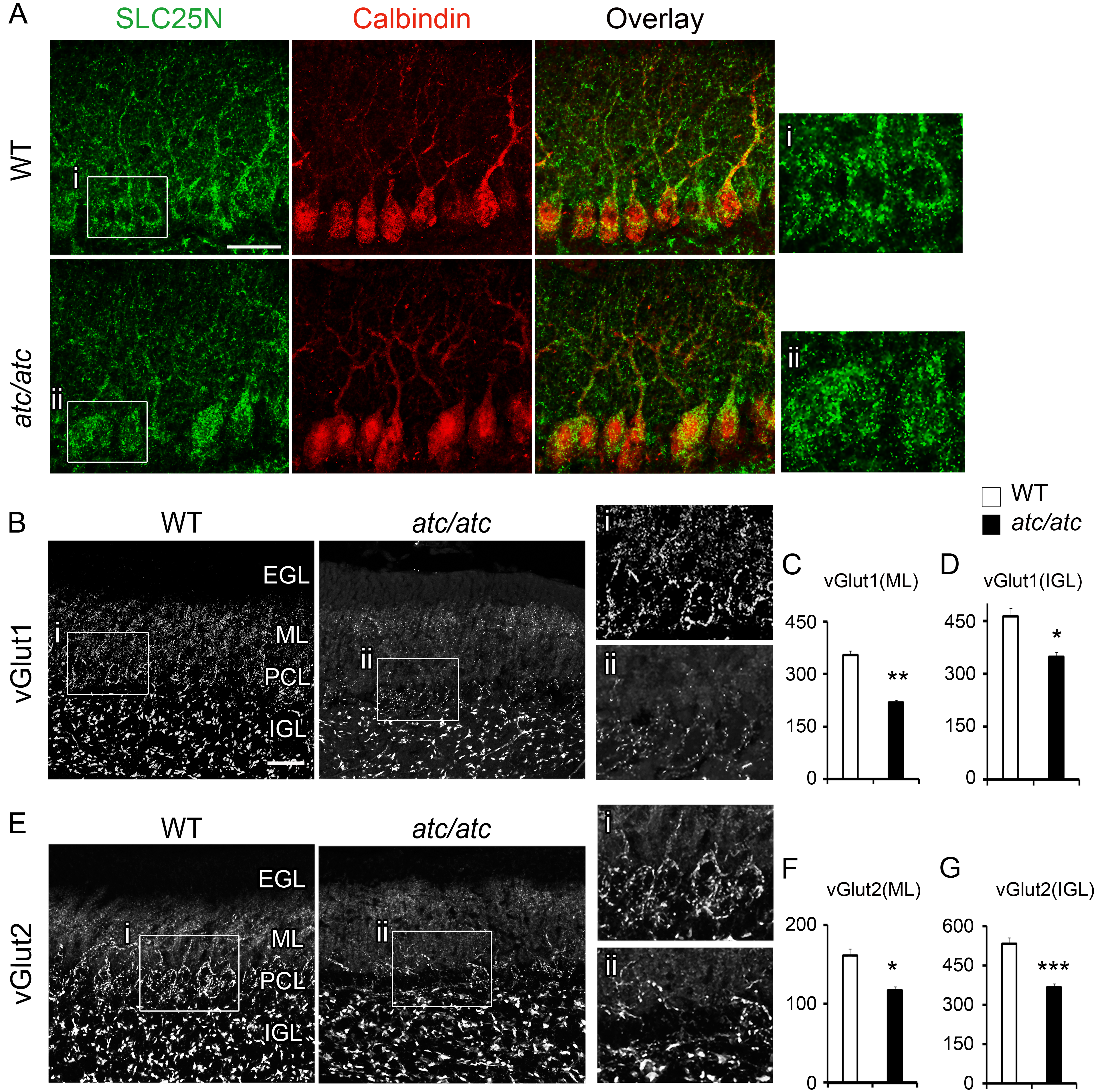

Supplement: S5 Fig — (A) Immunofluorescence labeling of P9 cerebellar mid-sagittal sections with SLC25A46, reveals diffused expression in the calbindin-positive PC somata of atc/atc mice. No differences were noted in the PC dendritic tree at this age. (B) Immunostaining of sagittal cerebellar sections reveals a significant decrease in the expression of vGlut1, both in the ML and the IGL of atc/atc mice. The insets are shown at higher power magnification. (C) Quantification of fluorescence intensity in the ML (p = 0.0028) and (D) in the IGL (p = 0.06) (n = 3 mice per genotype). (E) Immunostaining of sagittal cerebellar sections shows significant reduction in the expression of vGlut2, both in the ML and the IGL of atc/atc mice. (F) Quantification of fluorescence intensity in ML (p = 0.078) and (G) in the IGL (p = 0.0008) (n = 3 mice per genotype). Statistical significance was determined by two-tailed unpaired Student’s t-test. Data represent mean values ± SEM Scale bars, 40 μm. (TIF) [file pgen.1006656.s005.tif]

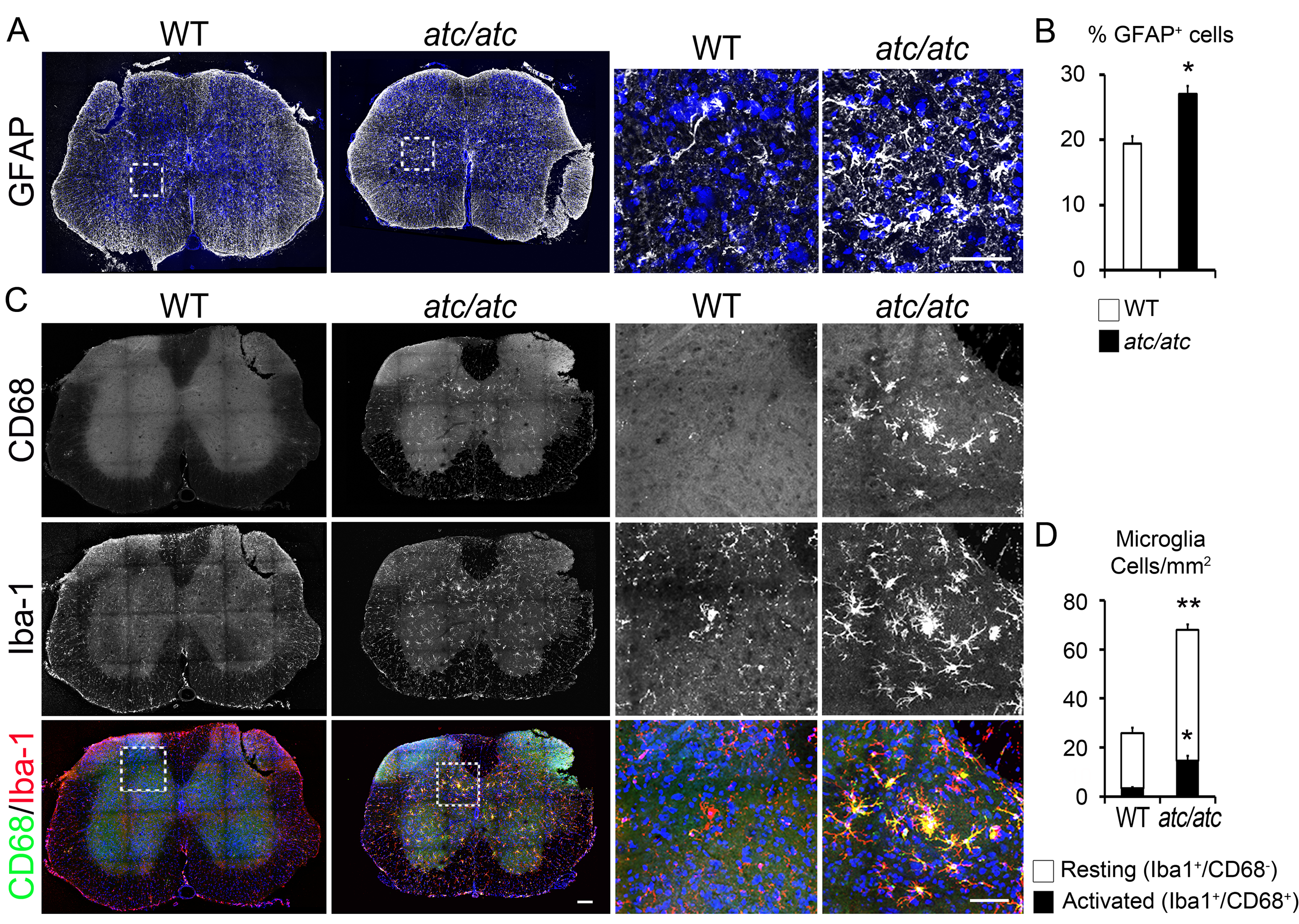

Supplement: S6 Fig — Confocal immunofluorescence images of transverse sections of the lower thoracic/lumbar spinal cord of WT and atc/atc littermates. (A) GFAP labeling (white) at low and the insets at high power micrographs. (B) Quantification of the percentage of GFAP+ cells showing a statistically significant increase in atc/atc (n = 3 mice per genotype, p = 0.019). (C) Confocal images of double immunofluorescence against CD68 (activated microglia/macrophages; green in merged images) and Iba-1 (total microglia/macrophages; red in merged images) in spinal cord sections of WT and atc/atc mice, at low and the insets at high power magnification. Scale bars, 100 μm and 50 μm for low and high power images, respectively. TO-PRO-3 (blue) shows nuclear counterstaining. (D) Quantification revealed a significant increase in the density of Iba1+/CD68- cells/ mm2 (resting microglia/macrophages, p = 0.002) and Iba1+/CD68+ cells/ mm2 (activated microglia/macrophages, p = 0.0284) in atc/atc mice as compared to the WT (n = 3 mice per genotype). * p≤0.05; ** p≤0.01; *** p≤0.001. Statistical significance was determined by two-tailed unpaired Student’s t-test. Data represent mean values ± SEM. (TIF) [file pgen.1006656.s006.tif]

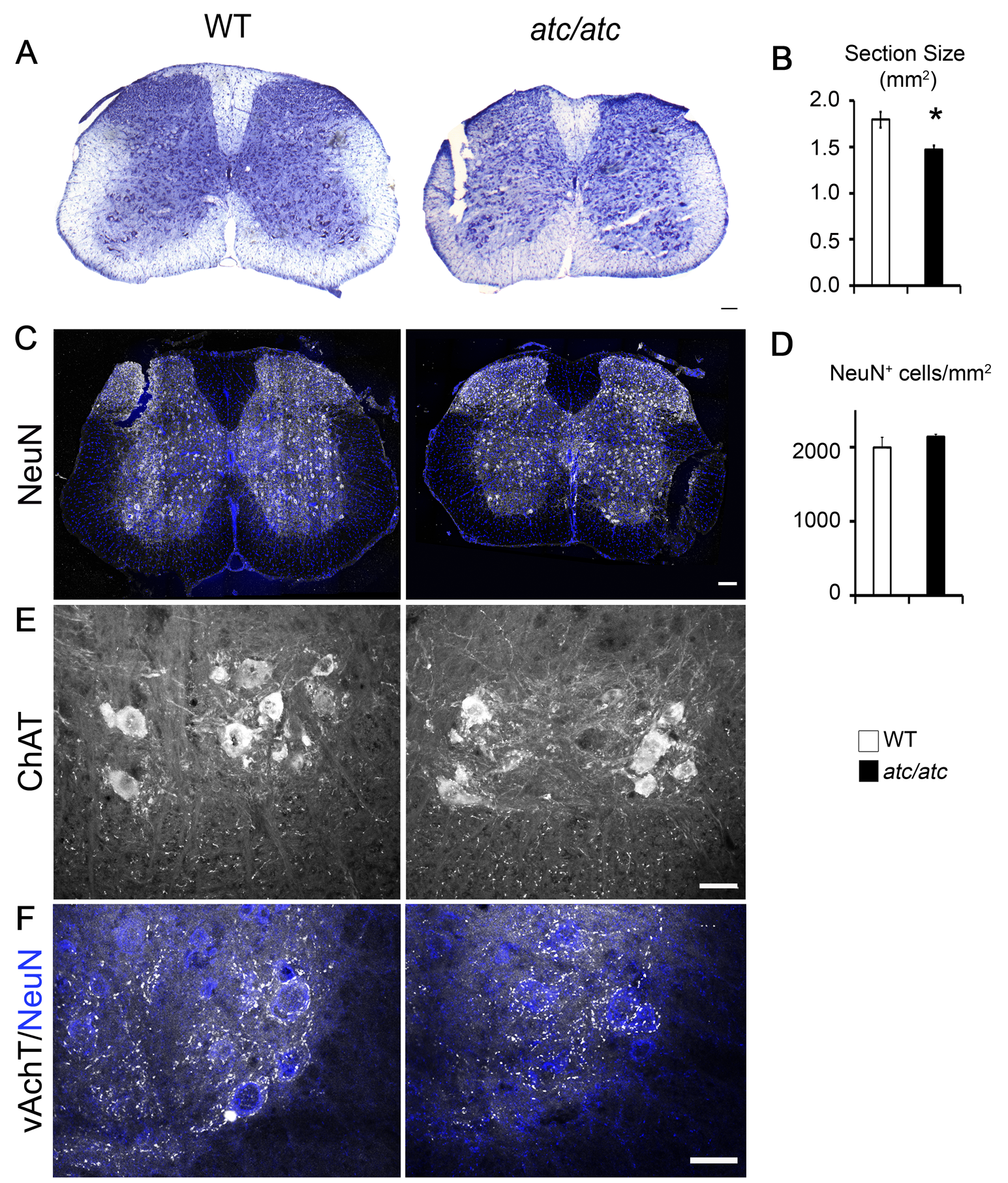

Supplement: S7 Fig — (A) Toluidine blue staining of spinal cord transverse sections at the lower thoracic/lumbar level; (B) Quantification of the area of equivalent, toluidine blue stained spinal cord sections verified the smaller size of the spinal cord as expected due to the reduced growth of atc/atc mice as compared to the WT (n = 3 mice per genotype, p = 0.041). Scale bars, 100 μm for A and C (low magnification); 40 μm for E and F. * p≤0.05. Statistical significance was determined by two-tailed unpaired Student’s t-test. Data represent mean values ± SEM Confocal immunofluorescence images of transverse sections of the lower thoracic/lumbar spinal cord of WT and atc/atc littermates. (C) NeuN labeling of neuronal cells (white) and TO-PRO-3 counter stain of nuclei (blue). (D) Quantification of the number of NeuN+ neurons per area of the spinal cord section (n = 3 mice per genotype, p = 0.415). (E) Confocal images of immunofluorescence against ChAT which labels the motor neurons that reside in the ventral horns of the spinal cord. (F) Double immunofluorescence against vAchT (white) and NeuN (blue) visualizes the cholinergic synapses on the motor neurons of the spinal cord ventral horns. (TIF) [file pgen.1006656.s007.tif]

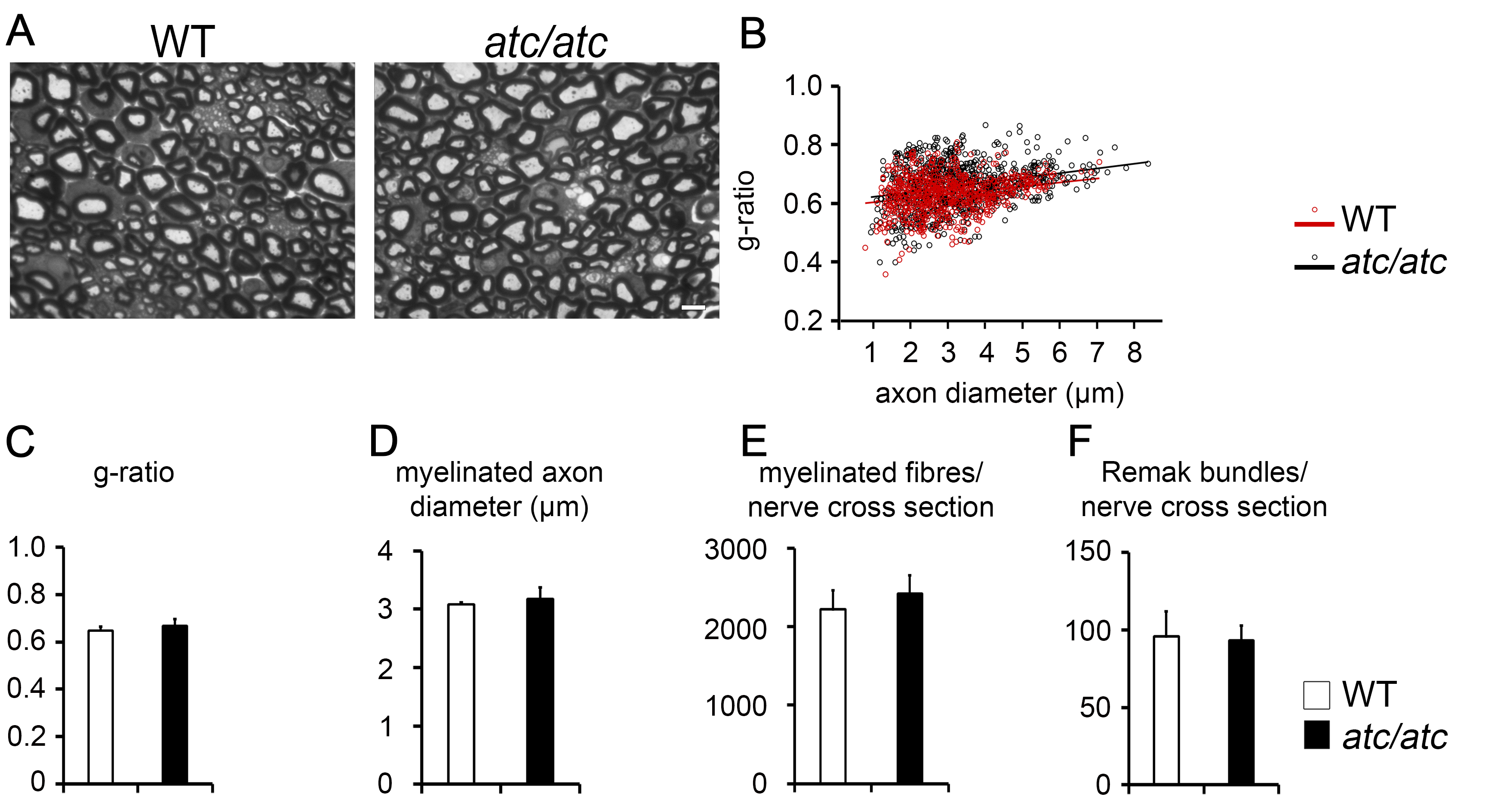

Supplement: S8 Fig — (A) Representative toluidine blue stained semithin cross sections of 4-week-old WT and atc/atc mice. Scale bar: 5 μm. (B) Scatter plot of g-ratio as a function of axon diameter indicating a similar distribution of values among WT and atc/atc fibers (n = 689 myelinated WT fibers and n = 905 myelinated atc/atc fibers pooled from 3 nerves per genotype). (C) Histogram representing mean values ± SEM of calculated g-ratios (n = 3 nerves per genotype; p = 0.621). Quantification of (D) the mean diameter of myelinated axons (n = 3 nerves per genotype; p = 0.711), (E) the number of myelinated axons per nerve (n = 3 nerves per genotype; p = 0.582) and (F) the number of Remak bundles of non-myelinated small diameter axons (n = 3 nerves per genotype; p = 0.908). Statistical significance was determined by two-tailed unpaired Student’s t-test. Data represent mean values ± SEM. (TIF) [file pgen.1006656.s008.tif]

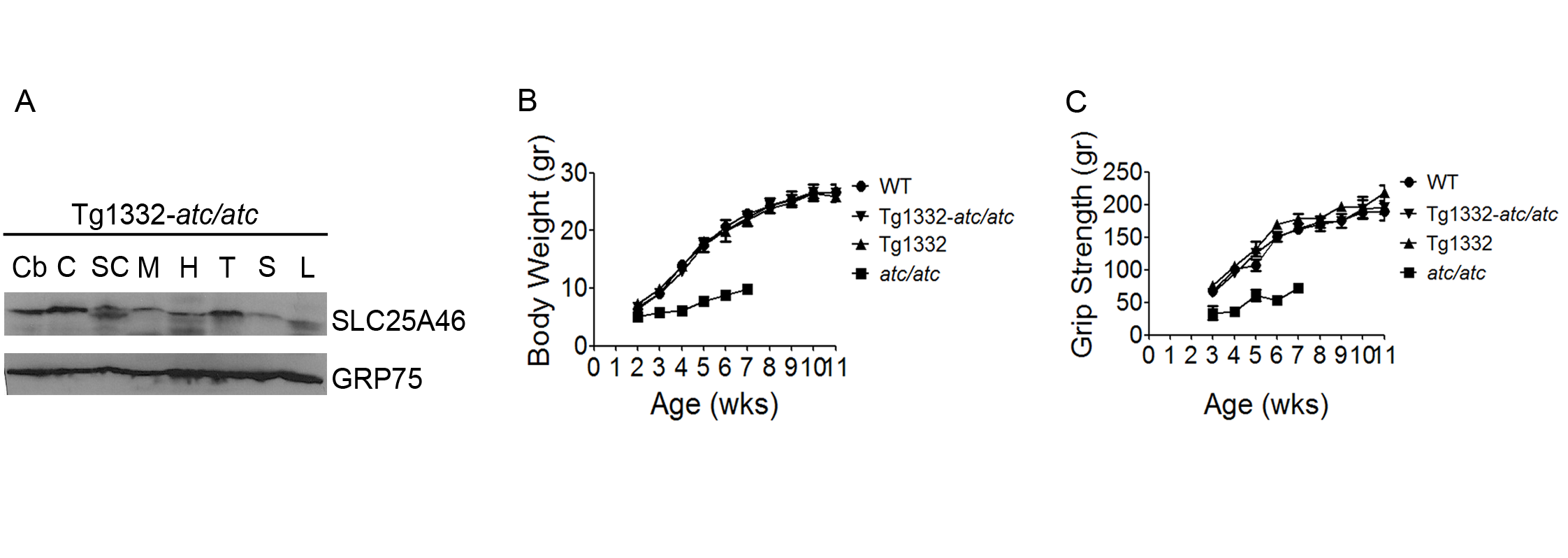

Supplement: S9 Fig — (A) Expression pattern of human SLC25A46 on mitochondrial extracts from cerebrum (B), cerebellum (C), spinal cord (SC), muscle (M), heart (H), thymus (T), spleen (S) and liver (L) tissues of TghuSLC25A46/Slc25a46atc/atc (Tg1332-atc/atc) mice through immunoblotting with antibodies against SLC25A46 and the mitochondrial protein GRP75. Complete rescue of (B) body weight gain and (C) muscle weakness in atc/atc mice expressing human SLC25A46 (Tg1332-atc/atc). All mice used were sex matched littermates(n = 6 per group). (TIF) [file pgen.1006656.s009.tif]
