# Supplementary material for: Novel insights into SLC25A46-related pathologies in a genetic mouse model
Source: PLoS Genet. 2017 Apr 4;13(4):e1006656. doi: 10.1371/journal.pgen.1006656 (PMC5380310; doi:10.1371/journal.pgen.1006656)
Supplement: S1 Table — SSLPs and SNPs are shown in the left column and respective chromosome 18 position (in bp) are shown in the right column. B, H and D denote homozygosity for mutagenesis strain, heterozygosity and homozygosity for the mapping strain, respectively. Bottom row shows numbers of individual mice. Red and black font denotes ataxic and not affected mice, respectively. All ataxic mice screened were found homozygous for markers D18Mit225, D18Mit60 and D18Mit158. Four ataxic mice were found heterozygous for D18Mit22 and one mouse was found heterozygous for rs30151517 thus defining the chromosomal position where the mutant locus lies. The two candidate region defining markers and their respective positions are shown in bold. (DOC) [file pgen.1006656.s010.doc]

| **MARKER** | **GENOTYPE** | | | | | | **POSITION** |
| --- | --- | --- | --- | --- | --- | --- | --- |
| D18Mit132 | B | B | H | D | D | B | 21,248,250 |
| D18Mit172 | B | B | H | D | D | B | 23,594,669 |
| **D18Mit22** | B | B | H | D | D | H | **24,953,907** |
| D18Mit225 | B | B | B | D | H | H | 30,615,771 |
| D18Mit60 | B | B | B | D | H | H | 32,489,109 |
| D18Mit158 | B | B | B | D | H | H | 33,486,280 |
| **rs30151517** | - | H | - | - | - | - | **33,795,698** |
| rs13483295 | - | H | B | - | - | - | 34,607,279 |
| D18Mit120 | B | H | H | D | H | H | 36,054,514 |
| D18Mit186 | H | H | H | D | B | B | 72,020,418 |
| DNA No | 181 | 188 | 190 | 443 | 444 | 445 |  |
